# Nutritional anemia is associated with increased risk of severe COPD exacerbations: a prospective cohort study

**DOI:** 10.1016/j.ajcnut.2026.101402

**Published:** 2026-06-20

**Authors:** Valerie Dehondt, Nicolas Kint, Lowie EGW Vanfleteren, Lies Lahousse

**Affiliations:** 1 Department of Bioanalysis, Ghent University, Ghent, Belgium; 2 Department of Hematology, Ghent University Hospital, Ghent, Belgium; 3 Department of Respiratory Medicine, Ghent University Hospital, Ghent, Belgium; 4 Department of Internal Medicine and Clinical Nutrition, Institute of Medicine, Sahlgrenska Academy, University of Gothenburg, Gothenburg, Sweden; 5 Department of Specialist Medicine, COPD Center, Sahlgrenska University Hospital, Gothenburg, Region Västra Götaland, Sweden; 6 Department of Epidemiology, Erasmus Medical Center, Rotterdam, The Netherlands

**Keywords:** chronic obstructive pulmonary disease, nutrition, anemia, exacerbations, mortality, hospitalization, iron deficiency, inflammation

## Abstract

**Background:**

Anemia prevalence in chronic obstructive pulmonary disease (COPD) varies widely across studies, and data on anemia subtypes and their association with mortality or COPD exacerbation readmission are limited.

**Objectives:**

We aimed to investigate anemia subtypes and their association with mortality and readmission for acute exacerbation of COPD (AECOPD) in patients hospitalized for AECOPD.

**Methods:**

In this prospective observational cohort study, patients with COPD aged ≥45 y hospitalized for severe exacerbations in a Belgian nationwide database were included. Cox regression models investigated associations between anemia (or its subtypes) and (postdischarge) mortality and AECOPD readmission, adjusted for age, sex, socioeconomic status, smoking, weight, and age-adjusted Charlson Comorbidity Index. Inverse probability of treatment weighting examined the association between anemia treatment and mortality across anemia subtypes.

**Results:**

Among 32,152 patients with COPD discharged from the hospital, 5956 (18.5%) had anemia. Nutritional anemia was most frequent (49.3%), followed by anemia of chronic disease (ACD; 31.3%). Anemia was associated with a 30% higher postdischarge mortality risk [adjusted hazard ratio (aHR): 1.30, 95% confidence interval (CI): 1.23, 1.37], and 8% higher readmission risk (aHR:1.08, 95% CI: 1.03, 1.14). Patients with hematologic anemia had the highest postdischarge mortality risk (aHR: 1.75, 95% CI: 1.54, 1.99), followed by nutritional anemia (aHR: 1.29, 95% CI: 1.20, 1.39). Only nutritional anemia was associated with increased readmission risk (aHR: 1.18, 95% CI: 1.10, 1.25). Treatment effects differed by anemia subtype, with iron, vitamin B12, and erythroid-stimulating agent (ESA) associated with lower postdischarge mortality in nutritional anemia; folic acid and iron in hematologic anemia; and ESA and iron in ACD.

**Conclusions:**

Nutritional anemia is the predominant subtype in hospitalized patients with AECOPD, and its increased readmission risk calls closer attention to iron deficiency in patients with COPD. All subtypes of anemia are associated with higher postdischarge mortality risk, but targeted treatment may reduce mortality risk and represents a potential strategy to improve outcomes.

## Introduction

Anemia is a chronic condition frequently associated with chronic obstructive pulmonary disease (COPD) and has gained attention as a comorbidity in recent years [1,2]. Its reported prevalence in patients with COPD varies widely from 5% to 38% [3]. Although COPD may also lead to secondary erythrocytosis through chronic hypoxia, anemia is more frequent [4]. Shared risk factors include systemic inflammation, older age, malnutrition, and oxidative stress [5,6]. Fatigue and dyspnea, key symptoms in COPD, are also associated with anemia [5].

Chronic anemia in COPD is likely multifactorial and may result from: *1*) chronic inflammation leading to anemia of chronic disease (ACD), with elevated inflammatory cytokines inducing hepcidin, which reduces intestinal iron absorption and increases iron sequestration in macrophages; these cytokines also impair erythropoietin production, damage erythroid progenitors, and shorten red blood cell (RBC) survival [5,7]; *2*) inadequate dietary nutrient intake [3], as iron deficiency is common in COPD and contributes to nutritional anemia [8]; and *3*) comorbidities, including hematological disorders such as myelodysplastic syndromes [9]. ACD, also known as anemia of inflammation, is considered the predominant mechanism in COPD [5].

Anemia has been associated with increased exacerbation risk, mortality, and reduced quality of life in COPD [10]. However, most studies have small sample sizes and do not fully account for comorbidities, for example, malignancy [11]. Moreover, studies on the effect of different anemia causes on exacerbations or mortality are lacking. Systemic inflammation, which contributes to anemia, often increases during exacerbations, thereby amplifying the impact of anemia on clinical outcomes [12]. Therefore, this nationwide cohort study aims to: *1*) investigate the prevalence of anemia and its subtypes in patients with COPD hospitalized for acute exacerbation, *2*) assess the association of anemia (subtypes) with (postdischarge) mortality and readmission for severe exacerbations, and *3*) investigate the association between anemia treatment and postdischarge mortality. We hypothesized that ACD would be associated with a higher risk of mortality and readmission, and that treatment of this anemia type would be associated with a lower risk of postdischarge mortality.

## Methods

### Study population

Patients with COPD aged ≥45 y were included in this Belgian nationwide observational cohort when having a hospitalized/severe exacerbation defined as hospital admission or emergency department visit with a primary diagnosis code of (acute) COPD exacerbation (AECOPD), COPD with acute lower respiratory infection [International Classification of Diseases, 10th Revision (ICD-10): J44.0 or J44.1], or with a primary diagnosis of chronic lower respiratory disease (ICD-10: J40-J47) or respiratory failure (ICD-10: J96), combined with a secondary diagnosis code for AECOPD or COPD with acute lower respiratory infection (ICD-10: J44.0 or J44.1), during follow-up from 1 January, 2017 to 1 January, 2022. Hospitalizations with a concomitant pneumonia diagnosis were excluded [13]. Discharge date of the first exacerbation was defined as the index date (Supplemental Figure 1). Follow-up ended at the occurrence of AECOPD readmission, death, emigration, or end of study, whichever came first. This study received approval from InterMutualistic Agency (IMA) and Minimal Hospital Dataset (MHD) database administrators and “Social Security and Health Chamber of the Belgian Information Security Committee” (approval code IVC/KSZG/23/008), waiving the need for individual informed consents [14]. Patients with missing data in key variables (age, sex) were excluded (limited to <0.1% of the source population, Supplemental Figure 2). The IMA databases represent all legal residents of Belgium, as health insurance is legally mandatory. Moreover, all Belgian hospitals are required to provide administrative, medical, and nursing data to the centralized MHD database [15,16]. Consequently, this dataset is complete, and no missing data were present for the covariates used in this study. The study was designed according to the STROBE guidelines (Supplemental Table 1). Details on the methodology have been published and are provided in Supplementary Materials (Supplemental Table 2) [13,17].

### Anemia subtypes

The exposure of interest was the presence of chronic anemia. Chronic anemia was identified by ICD-10 codes (D46.0-D46.4, D50-D53, D55-D61, D63-D64) recorded during the year before the index date, which previously showed high positive predictive values for anemia subtypes based on the Danish National Patient Registry [18,19]. Patients were classified into mutually exclusive subtypes: hematologic anemia (hemolytic: D55-D59; aplastic: D61, D64.81; refractory/myelodysplastic: D46.0-D46.4, D64.0-D64.2), ACD (D63), and nutritional anemia (D50-D53). Patients with unspecified/other anemia (D64.89, D64.9) were classified by comorbidities based on literature, in sequential order: those with specific blood cancers and myelodysplastic syndrome as hematologic anemia [20]; those with other cancers, HIV, respiratory tuberculosis, autoimmune diseases (rheumatoid arthritis, systemic lupus erythematosus, connective-tissue diseases, vasculitis, sarcoidosis, inflammatory bowel disease), chronic rejection after solid-organ transplantation, or chronic kidney disease (CKD) as ACD [21]; and those with malnutrition as nutritional anemia (Supplemental Table 2). Patients without any of these diagnoses remained in the unspecified/other anemia category.

### Mortality and readmission

The primary outcome of interest was time to postdischarge mortality. The secondary outcome was time to first AECOPD readmission, defined using the same ICD-10 coding criteria as those applied for cohort inclusion, with the admission date as the incident date of readmission [13]. Finally, we analyzed in-hospital mortality as a supplementary outcome in the population hospitalized for AECOPD.

### Covariates

Models were adjusted for age, sex, socioeconomic status (SES), smoking history, weight, and age-adjusted Charlson Comorbidity Index (CCI; excluding chronic pulmonary disease in the calculation). SES was determined from medical coverage, with low SES defined as increased medical reimbursements provided to individuals with low income. Patients were categorized as (ever-)smoking if any ICD code [e.g., (history of) nicotine dependence] (ICD-9 until 2014, ICD-10 since 2015), anatomical therapeutic chemical (ATC) code (N07BA, N06AX12: drugs used in nicotine dependence), or medical procedure code (e.g., tobacco counseling) was recorded between 1 January, 2010 and the index date. Weight was categorized as underweight or overweight using ICD and medical procedure codes from the year before the index date, or as normal in lack thereof. Anemia treatment [iron, vitamin B12, folic acid, and erythroid-stimulating agents (ESAs)] was assessed during the year before the index date using ATC codes (Supplemental Table 2).

### Statistical analyses

Cause-specific Cox proportional hazard analyses were used to estimate hazard ratios (HRs) with 95% confidence intervals (CIs) for the association between ICD-coded anemia (compared with no anemia) and postdischarge outcomes among patients alive at discharge. For postdischarge mortality analyses, persons were censored at AECOPD readmission, emigration, or the end of the study. For AECOPD readmission analyses, death during follow-up was treated as a competing event by censoring patients at the time of death. Analyses included a crude model, followed by adjustment for demographics [age, sex, SES, (ever-)smoking, weight; model 1], and CCI (model 2). For anemia subtypes (hematologic anemia, ACD, nutritional anemia, other) (compared with no anemia), the same models were applied. Furthermore, to examine whether anemia treatment modified postdischarge mortality, interaction terms between anemia subtypes and anemia treatment were included. To address confounding by indication, inverse probability of treatment weighting (IPTW) was performed. Propensity scores (PS) were estimated separately for each treatment using logistic regression models incorporating demographic variables, comorbidities, markers of disease severity, and other anemia-related treatments. On the basis of PS, weights were calculated to estimate the average treatment effect of the treated and were trimmed at the 0.5th and 99.5th percentiles. Standardized mean differences for each covariate were calculated to evaluate covariate balance before and after weighting, with a threshold of ≥0.1 to indicate imbalance between treatment groups, and were graphically presented in Love plots (Supplemental Figure 3). Subsequently, weighted Cox proportional hazards analyses were applied to estimate adjusted HRs (aHRs) with 95% CIs, using robust variance estimators and including interaction terms between anemia subtypes and the corresponding treatment.

The proportional hazards assumption was assessed using visual inspection of the scaled Schoenfeld residual plots and formal Schoenfeld residual tests. No multicollinearity was observed.

Logistic regression analyses estimated odds ratios (ORs) with 95% CI for the association between anemia of any type (compared with no anemia) and in-hospital mortality, and between anemia subtypes and in-hospital mortality, using the same adjusted models.

A 2-sided *P* value <0.0167 after Bonferroni correction for multiplicity was considered statistically significant for the primary outcomes. All analyses were performed in R (R version 4.3.0).

### Sensitivity analyses

For postdischarge mortality analyses, comorbidities (frailty, cancer, malnutrition, CKD, heart failure, diabetes mellitus, arrhythmia, and hypertension) were included individually instead of CCI, and were identified in the year before the index date using ICD-coded diagnoses, medical procedure codes, and/or medication use (Supplemental Table 2). Frailty was defined based on the John Hopkins Claims-based Frailty Indicator, with ≥0.20 defined as frail persons [22]. To assess the robustness of the comorbidity-based reclassification of other/unspecified anemia, analyses were repeated without applying this reclassification and, in addition, after excluding patients with other or unspecified anemia, thereby restricting analyses to clearly defined anemia subtypes only. For AECOPD readmission analyses, model 2 analyses were repeated using Fine–Gray subdistribution hazard models, treating death as a competing event.

## Results

### Baseline characteristics

A total of 34,864 patients were hospitalized for a severe AECOPD, of which 2712 (7.8%) patients died during hospitalization. The study population alive at discharge included 32,152 patients (Supplemental Figure 2), of which 5956 (18.5%) had a diagnosis of anemia. Patients with anemia were more often male, older, of lower SES, underweight or overweight, and had more comorbidities. Among anemic patients, 2939 (49.3%) had nutritional anemia, 1864 (31.3%) had ACD, 557 (9.4%) had hematologic anemia, and 596 (10.0%) had other types. Among patients with nutritional anemia, 1923 (65.4%) had iron deficiency anemia (IDA). All variables differed significantly between the subtypes (*P* <0.001). The 4 most important differences were that females were more represented in nutritional and other anemia types than in hematologic anemia or ACD, patients with ACD were older, more often overweight, and had fewer (ever-)smokers than those with other anemia subtypes (Table 1).

**TABLE 1:** Baseline characteristics of hospitalized patients with AECOPD (*N* = 32,152) according to anemia status and subtype

	Total (*N* = 32,152)	No anemia (*n* = 26,196)	Anemia (*n* = 5956)	Anemia subtypes			
				Hematologic anemia (*n* = 557)	ACD (*n* = 1864)	Nutritional anemia (*n* = 2939)	Other types of anemia (*n* = 596)
Demographics							
Female, *n* (%)	14,593 (45.4)	12,028 (45.9)	2565 (43.1)	211 (37.9)	722 (38.7)	1345 (45.8)	287 (48.2)
Age in y (SD)	71.7 (10.4)	71.0 (10.4)	74.9 (10.0)	73.2 (9.7)	77.1 (9.5)	74.3 (10.2)	72.9 (9.8)
Low SES, *n* (%)	14,970 (46.6)	11,926 (45.5)	3044 (51.1)	262 (47.0)	954 (51.2)	1528 (52.0)	300 (50.3)
(Ever-)smoker, *n* (%)	28,358 (88.2)	23,189 (88.5)	5169 (86.8)	487 (87.4)	1572 (84.3)	2585 (88.0)	525 (88.1)
Weight, *n* (%)							
Underweight	3414 (10.6)	2510 (9.6)	904 (15.2)	66 (11.8)	236 (12.7)	536 (18.2)	66 (11.1)
Normal weight	21,200 (65.9)	17,772 (67.8)	3428 (57.6)	339 (60.9)	1056 (56.7)	1638 (55.7)	395 (66.3)
Overweight	7538 (23.4)	5914 (22.6)	1624 (27.3)	152 (27.3)	572 (30.7)	765 (26.0)	135 (22.7)
Comorbidities							
CCI (IQR)	5 (3–7)	4 (3–6)	7 (5–9)	8 (6–10)	8 (7–10)	6 (4–8)	4 (3–6)
Frailty, *n* (%)	9184 (28.6)	6364 (24.3)	2820 (47.3)	206 (37.0)	1052 (56.4)	1359 (46.2)	203 (34.1)
Cancer, *n* (%)	5342 (16.6)	3663 (14.0)	1679 (28.2)	389 (69.8)	710 (38.1)	580 (19.7)	0
Malnutrition, *n* (%)	5409 (16.8)	3508 (13.4)	1901 (31.9)	179 (32.1)	582 (31.2)	1140 (38.8)	0
Chronic kidney disease, *n* (%)	6680 (20.8)	4307 (16.4)	2373 (39.8)	208 (37.3)	1321 (70.9)	844 (28.7)	0
Heart failure, *n* (%)	10,485 (32.6)	7470 (28.5)	3015 (50.6)	267 (47.9)	1072 (57.5)	1465 (49.8)	211 (35.4)
Diabetes mellitus, *n* (%)	11,065 (34.4)	8247 (31.5)	2818 (47.3)	260 (46.7)	970 (52.0)	1355 (46.1)	233 (39.1)
Arrhythmia, *n* (%)	10,360 (32.2)	7511 (28.7)	2849 (47.8)	267 (47.9)	1000 (53.6)	1370 (46.6)	212 (35.6)
Hypertension, *n* (%)	25,167 (78.3)	19,934 (76.1)	5233 (87.9)	462 (82.9)	1710 (91.7)	2567 (87.3)	494 (82.9)
Anemia treatment							
Iron, *n* (%)	3392 (10.5)	1318 (5.0)	2074 (34.8)	134 (24.1)	573 (30.7)	1283 (43.7)	84 (14.1)
Vitamin B12, *n* (%)	474 (1.5)	252 (1.0)	222 (3.7)	31 (5.6)	48 (2.6)	132 (4.5)	11 (1.8)
Folic acid, *n* (%)	4290 (13.3)	2740 (10.5)	1550 (26.0)	160 (28.7)	432 (23.2)	869 (29.6)	89 (14.9)
Erythroid-stimulating agents, *n* (%)	604 (1.9)	222 (0.8)	382 (6.4)	84 (15.1)	251 (13.5)	47 (1.6)	0

All *P* values comparing patients with and without anemia were <0.001, based on χ^2^ tests for categorical variables, *t*-tests for normally distributed continuous variables, and Mann-Whitney *U* tests for nonnormally distributed continuous variables.

During the reclassification process, patients with unspecified or other anemia (D64.89, D64.9) and concomitant cancer or chronic kidney disease were classified as having anemia of chronic disease, whereas those with malnutrition were classified as having nutritional anemia. Consequently, these comorbidities are not present in the “Other types of anemia” group.

Abbreviations: ACD, anemia of chronic disease; AECOPD, acute exacerbations of chronic obstructive pulmonary disease; CCI, Charlson Comorbidity Index; SES, socioeconomic status.

### Postdischarge mortality

**Figure 1:**
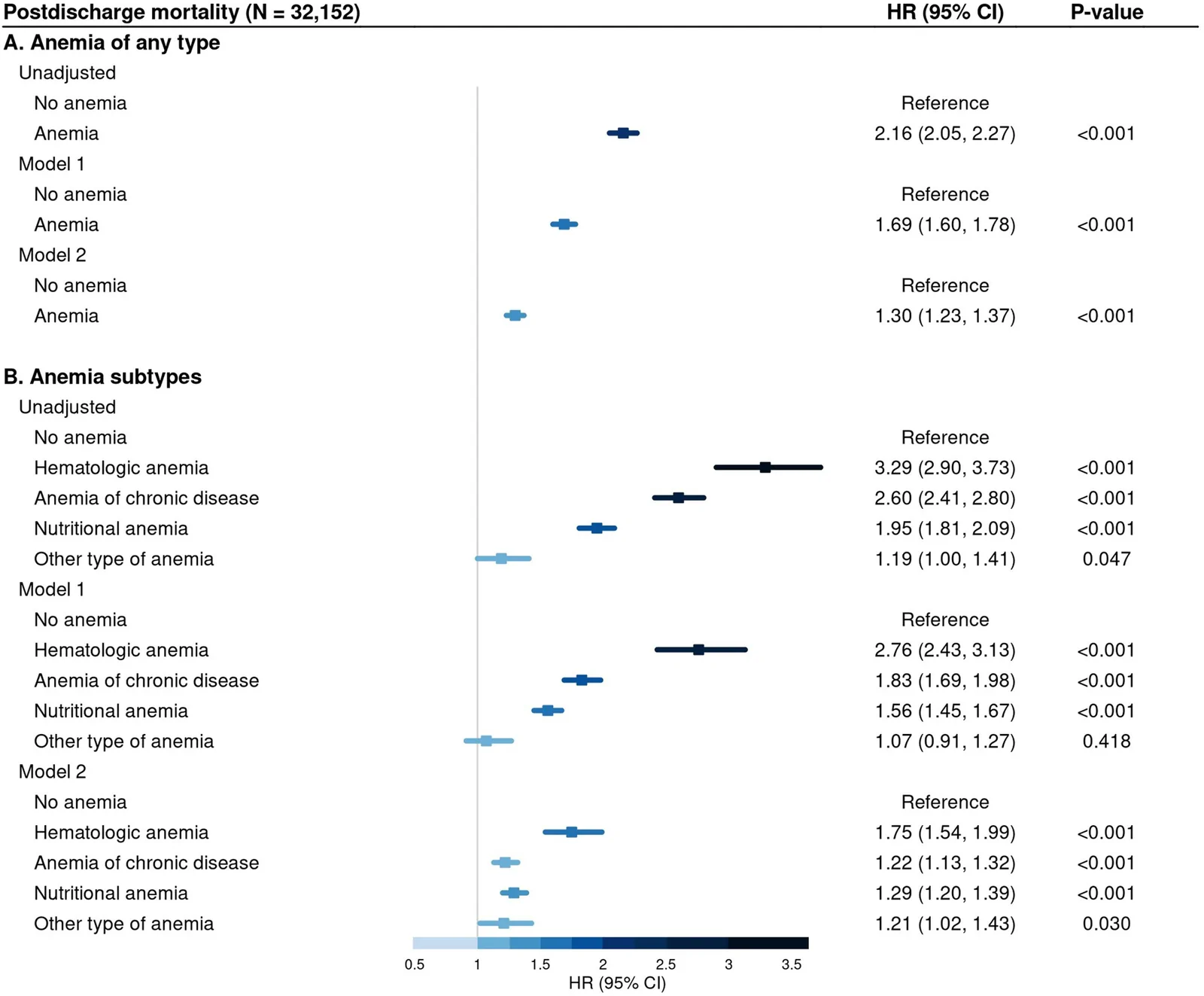
Hazard ratios for the association between anemia and postdischarge mortality in patients with COPD hospitalized for COPD exacerbation. (A) Anemia of any type. (B). Anemia classified into subtypes. Model 1: adjusted for demographics [age, sex, SES, (ever-)smoking, and weight]. Model 2: model 1 + adjustment for age-adjusted Charlson Comorbidity Index. CI, confidence interval; COPD, chronic obstructive pulmonary disease; HR, hazard ratio; SES, socioeconomic status.

**Figure 2:**
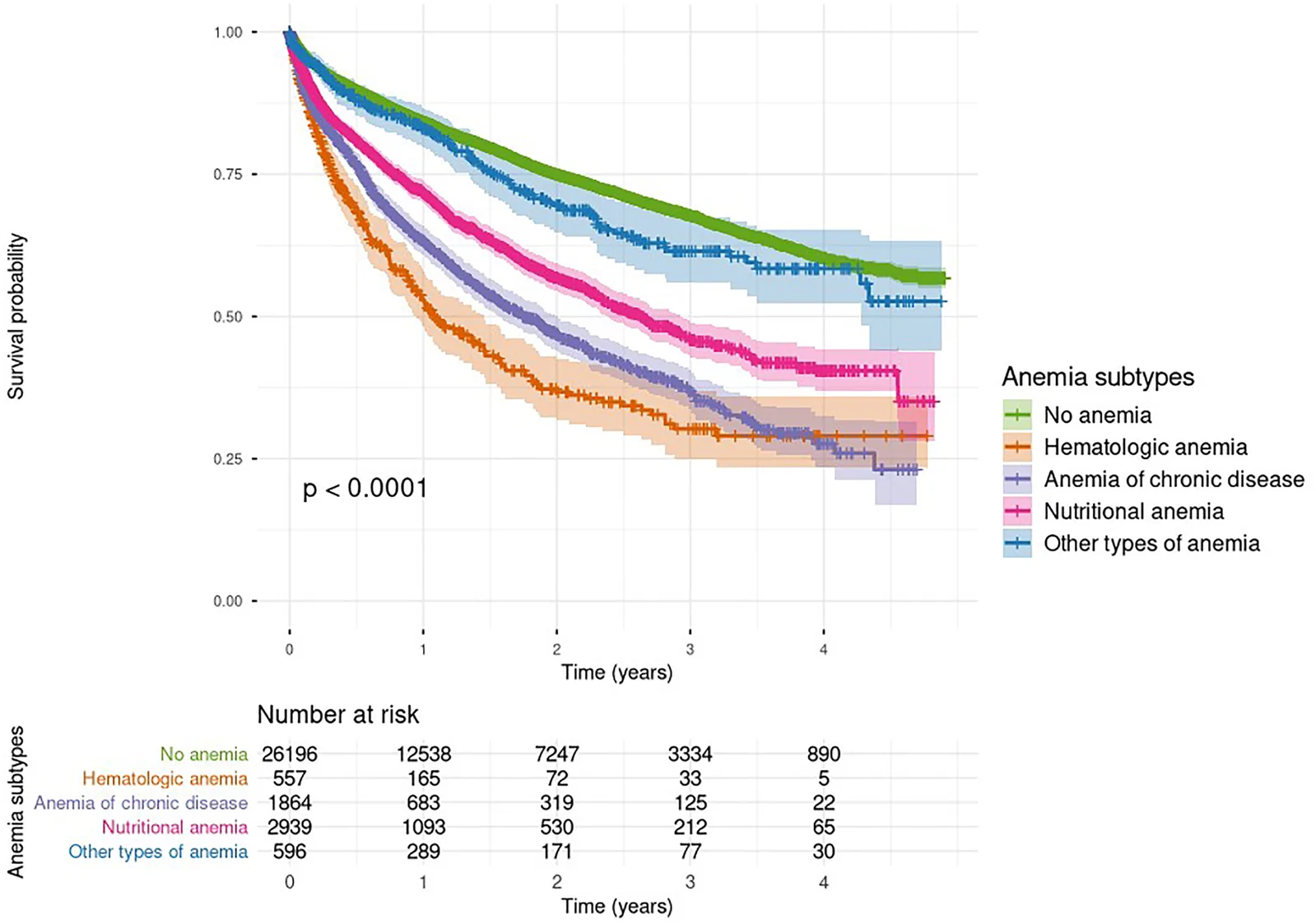
Kaplan–Meier curves with risk table for postdischarge survival in patients with and without anemia, stratified by anemia subtype.

Among discharged patients with COPD, 7189 (22.4%) patients died during a median follow-up of 0.8 y (IQR 0.2- 2.0; 40,883 persons-years), taking censoring at readmission into account, of whom 2052 (28.5%) had anemia. The Kaplan–Meier curves for anemic and nonanemic patients are shown in Supplemental Figure 4. Patients with anemia had a 69% higher postdischarge mortality risk after a severe AECOPD than those without anemia, adjusted for demographics (Figure 1A–model 1: aHR: 1.69, 95% CI: 1.60, 1.78). This association remained significant after further adjustment for CCI (Figure 1A–model 2: aHR: 1.30, 95% CI: 1.23, 1.37), corresponding to an additional 30% higher postdischarge mortality risk in patients with anemia beyond the effect of CCI. The Kaplan–Meier survival curves for patients with and without anemia, stratified by anemia subtype, are shown in Figure 2. After adjusting for demographics, hematologic anemia was associated with a 2.76-fold higher postdischarge mortality risk (aHR: 2.76, 95% CI: 2.43, 3.13), ACD and nutritional anemia with an 83% and 56% higher risk, respectively (aHR: 1.83, 95% CI: 1.69, 1.98; aHR: 1.56, 95% CI: 1.45, 1.67), whereas other types showed no significant association (aHR: 1.07, 95% CI: 0.91, 1.27) compared with patients without anemia (Figure 1B, model 1). After adjustment for CCI, hematologic anemia, ACD, and nutritional anemia remained significantly associated with higher mortality risk (Figure 1B–model 2: hematologic anemia: aHR: 1.75, 95% CI: 1.54, 1.99; ACD: aHR: 1.22, 95% CI: 1.13, 1.32; nutritional anemia: aHR: 1.29, 95% CI: 1.20, 1.39). When incorporating interaction terms between anemia subtypes and treatment in IPTW analyses, iron was significantly associated with reduced postdischarge mortality in nutritional anemia (aHR: 0.65, 95% CI: 0.55, 0.78), in hematologic anemia (aHR: 0.68, 95% CI: 0.49, 0.95), and in ACD (aHR: 0.81, 95% CI: 0.67, 0.98); vitamin B12 in nutritional anemia (aHR: 0.65, 95% CI: 0.45, 0.95); folic acid in hematologic anemia (aHR: 0.62, 95% CI: 0.45, 0.86); and ESA treatment in ACD (aHR: 0.63, 95% CI: 0.46, 0.85), and in nutritional anemia (aHR: 0.56, 95% CI: 0.32, 0.97) (Supplemental Tables 3–6).

### Readmission

**Figure 3:**
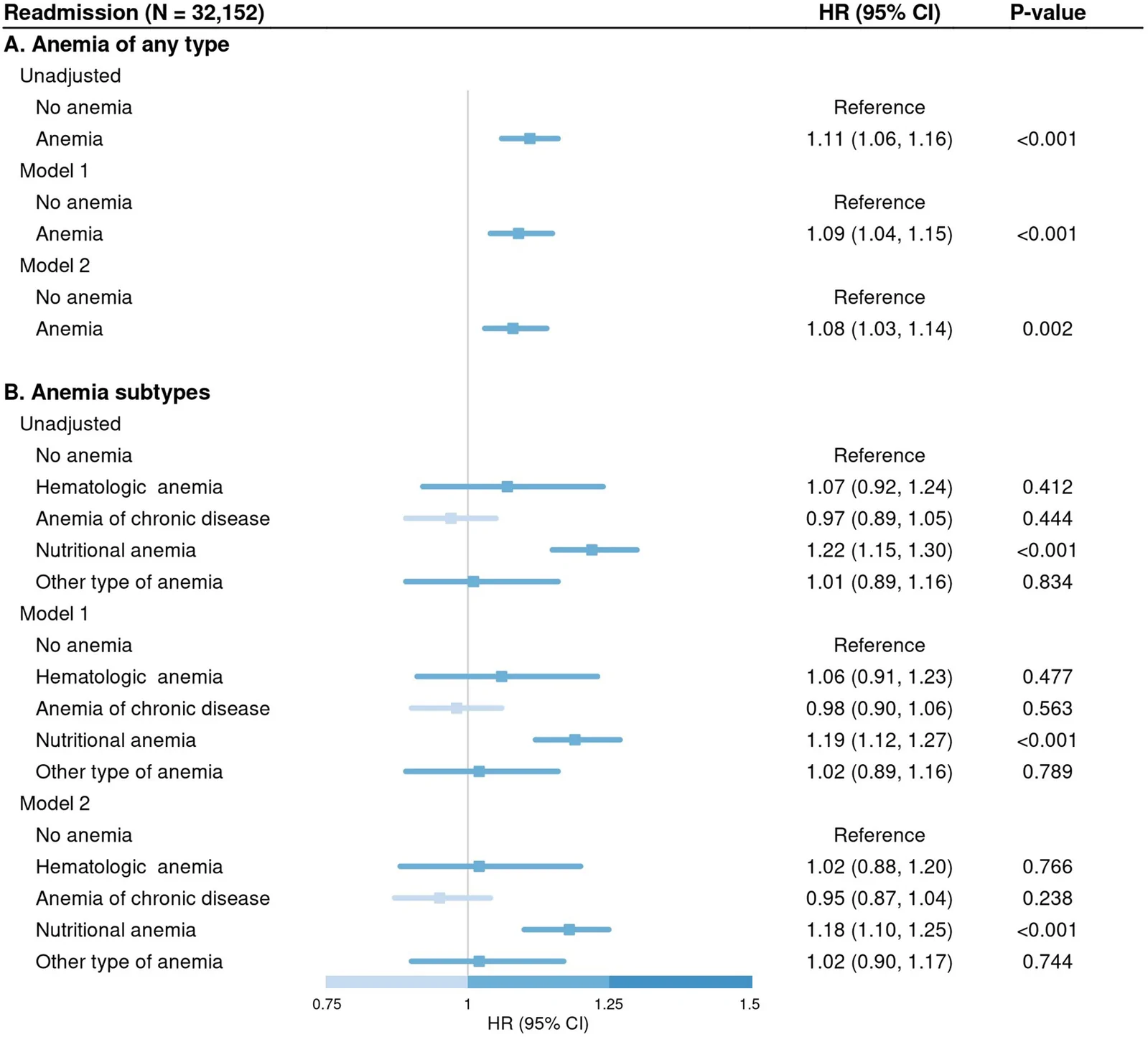
Hazard ratios for the association between anemia and readmission for severe exacerbation in patients with COPD hospitalized for COPD exacerbation. (A) Anemia of any type. (B) Anemia classified into subtypes. Model 1: adjusted for demographics [age, sex, SES, (ever-)smoking, and weight]. Model 2: model 1 + adjustment for age-adjusted Charlson Comorbidity Index. CI, confidence interval; COPD, chronic obstructive pulmonary disease; HR, hazard ratio; SES, socioeconomic status.

Among discharged patients, 11,965 (37.2%) were readmitted for a severe AECOPD during follow-up. After adjustment for demographics, anemia was associated with a 9% higher readmission risk (Figure 3A–model 1: aHR: 1.09, 95% CI: 1.04, 1.15), which remained significant after adjusting for CCI (Figure 3A–model 2: aHR: 1.08, 95% CI: 1.03, 1.14). When anemia was classified into subtypes, only nutritional anemia was significantly associated with a 19% higher readmission risk compared with no anemia after adjusting for demographics (Figure 3B–model 1: aHR: 1.19, 95% CI: 1.12, 1.27), which remained significant after adjusting for CCI (Figure 3B–model 2: aHR: 1.18, 95% CI: 1.10, 1.25). Other subtypes were not significantly associated with readmission.

### In-hospital mortality

Regarding the 7.8% patients who died during hospitalization, in-hospital mortality was higher among patients with anemia (750, 11.2%) than among those without anemia (1962, 7.0%). Patients with COPD with anemia had a 35% higher odds of in-hospital mortality than those without anemia, after adjusting for demographics [Supplemental Figure 5A–model 1: adjusted OR (aOR): 1.35, 95% CI: 1.23, 1.48]. However, this association lost significance after adjusting for CCI (Supplemental Figure 5A–model 2: aOR: 1.06, 95% CI: 0.97, 1.17). When categorizing anemia into subtypes, none were significantly associated with in-hospital mortality after adjusting for demographics and CCI (Supplemental Figure 5B–model 2).

### Sensitivity analyses

When comorbidities were included individually instead of CCI, postdischarge mortality remained significantly increased compared with no anemia (anemia of any type: aHR: 1.28, 95% CI: 1.21, 1.35; hematologic anemia: aHR: 1.80, 95% CI: 1.58, 2.06; ACD: aHR: 1.24, 95% CI: 1.14, 1.34; nutritional anemia: aHR: 1.23, 95% CI: 1.14, 1.32) (Supplemental Figure 6). Repeating analyses without reclassification (Supplemental Figures 7–9) and further restricting analyses to clearly defined anemia subtypes (Supplemental Figures 10–12) resulted in similar results. Fine–Gray analyses confirmed that only nutritional anemia was associated with a higher AECOPD readmission risk (Supplemental Figure 13).

## Discussion

In this real-world nationwide cohort of 32,152 patients with AECOPD discharged alive, 18.5% had a diagnosis of anemia. After adjustment for demographics and CCI, anemia was associated with 30% higher postdischarge mortality risk and 8% higher readmission risk. Nutritional anemia was the most prevalent subtype, followed by ACD and hematologic anemia. Only nutritional anemia was significantly associated with higher AECOPD readmission risk. However, all subtypes were significantly associated with a higher postdischarge mortality risk, with hematologic anemia showing the highest risk. Importantly, treatment with iron, vitamin B12, and ESA in nutritional anemia, ESA and iron in ACD, and folic acid and iron in hematologic anemia were associated with lower mortality risk. These findings underscore that addressing postdischarge mortality risk in patients with COPD requires multimorbidity management [1], and that anemia therapy is preferably subtype-specific.

Reported anemia prevalence in COPD varies widely, reflecting differences in cohort characteristics such as COPD severity and comorbidities [3]. A large United States study (>3 million patients with AECOPD) reported a prevalence of 17.0% [23], similar to our 18.5%. Contrastingly, a Chinese observational study reported 24.9% hospitalized patients with AECOPD had anemia [24], likely reflecting differences in study design, such as exclusion of specific comorbidities, restriction to tertiary care, and inclusion of patients who died during hospitalization, consistent with our observed trend toward higher in-hospital mortality among anemic patients. A Danish retrospective cohort of admitted patients with AECOPD reported a prevalence of 35.2%. The patients in this study were older (74.0 compared with 71.7 y), possibly explaining the higher prevalence [11].

Our study showed a 69% higher postdischarge mortality risk in patients with anemic AECOPD, which attenuated to 30% after additional adjustment for CCI. Similarly, the Danish study reported a 60% higher 1-y mortality risk in anemic hospitalized patients with AECOPD, but only adjusted for comorbidity count, besides age and sex [11]. Park et al. [25] found a similar HR of 1.31 after adjustment for demographics and CCI.

Anemia was significantly associated with an 8% higher AECOPD readmission risk in our study. A Spanish retrospective cohort study reported a 25% higher 30-d readmission risk in patients with anemic AECOPD, although without adjustment for key covariates such as smoking history, SES, and weight [26]. The Danish study also reported a 24% higher 1-y AECOPD readmission risk with limited adjustment [11]. Moreover, the COPDGene study reported a 63% higher rate of severe exacerbations, indicating more frequent severe exacerbations in anemic patients [27].

Anemia in COPD is associated with impaired cardiac function [28]. Reduced RBCs and oxygen delivery lower blood viscosity and peripheral vascular resistance, with compensatory vasodilatation, increased preload, and elevated cardiac output [28,29]. This may result in ventricular hypertrophy and myocardial remodeling [28,29], contributing to increased mortality [30]. Moreover, systemic inflammation and hypoxemia during exacerbations further stress the heart, increase pulmonary arterial pressure, and cardiovascular disease risk [31]. In our study, all anemia subtypes were associated with increased postdischarge mortality risk, with hematologic anemia showing the highest risk, likely reflecting underlying conditions such as malignancy. In nutritional anemia, iron therapy was associated with reduced postdischarge mortality, consistent with studies linking iron therapy to symptom improvement [27,32]. Iron deficiency is highly prevalent in COPD, likely driven by reduced intake, chronic inflammation, and comorbidities causing iron loss or malabsorption [33]. COPD is characterized by increased oxidative stress, which contributes to disease progression, exacerbations, and comorbidities [34], thereby increasing mortality risk [35]. In addition, previous research suggests that iron supplementation, beyond correcting anemia, may improve redox balance in iron-deficient patients with COPD, potentially mitigating oxidative stress [36,37]. These findings suggest that targeted treatment may mitigate mortality risk in specific anemia subtypes, although further research is needed.

Only nutritional anemia, predominantly IDA, was significantly associated with higher readmission risk. This aligns with a prospective cohort study linking iron deficiency to COPD exacerbations [33]. Iron supports energy metabolism and immune function [33]. Deficiency impairs mitochondrial and enzymatic activity, reduces skeletal and respiratory muscle performance, leading to lower physical activity, and increases susceptibility to infections and inflammation [33,36], potentially contributing to higher readmission risk in patients with COPD with nutritional anemia [38].

To our knowledge, this is the first study analyzing the association of mutually exclusive (separate) anemia subtypes on mortality and readmission in hospitalized patients with AECOPD. Nutritional anemia accounted for half of the anemic population, and ACD for one-third. Contrastingly, ACD is often reported as the most common subtype in COPD, reflecting the impact of inflammation [36]. However, anemia in inflammatory diseases is complex and multifactorial [39]. It is difficult to determine, as ACD is often misclassified as IDA, which we categorized as nutritional anemia, and often remains a diagnosis of exclusion [21]. Chronic inflammation, characteristic of COPD, elevates hepcidin, which reduces iron absorption and restricts iron release from macrophages and hepatocytes [36,40], leading to functional iron deficiency and ACD [10,36,40]. Contrastingly, absolute iron deficiency characterizes IDA [10]. We defined anemia subtypes as mutually exclusive categories to allow clear comparison. However, malnutrition and inflammation often coexist in clinical practice and may reinforce each other, leading to poorer outcomes [24].

Strengths of this study include the large sample size enabled by compulsory health insurance, reducing selection and recall bias, and the long-term follow-up. Both ambulatory and hospital data were used to define characteristics and comorbidities. We adjusted for a broad range of characteristics and comorbidities. Some limitations should be noted. First, coding errors and misclassification may occur, as anemia was defined using ICD codes because hemoglobin concentrations were unavailable. This may have led to underdiagnosis of mild anemia. Consequently, the observed associations may primarily reflect clinically recognized, more severe anemia, whereas underdiagnosis of mild anemia in the control group may have reduced the observed difference between anemic and nonanemic patients. In addition, identifying the anemia subtype was not always feasible, as anemia was often reported unspecified. Moreover, anemia can be multifactorial and may be attributable to other underlying factors despite using all available comorbidity information. However, the consistency of results across analyses with and without reclassification, or with or without excluding unspecified anemia, supports the robustness of our findings. For other important parameters, such as weight, we mitigated potential misclassification by broadly defining covariates using ICD codes, medical procedure codes, and medication use. Despite the comprehensive approach used to ascertain smoking history, some degree of misclassification cannot be entirely excluded. Future prospective studies should incorporate laboratory parameters, for example, hemoglobin, measured at AECOPD diagnosis and monitored as a continuous variable alongside anemia subtype, to validate ICD-based anemia classification and further elucidate underlying biological mechanisms. Second, as in all observational studies, causality cannot be proven, and residual confounding may remain, despite IPTW-adjusted analyses. Although the influence of unmeasured factors related to anemia severity cannot be excluded, results confirm avoiding the use of anemia therapy when not indicated in patients with COPD, given their potential for iron overload and increasing oxidative stress or infection risk in this exacerbation-prone population [41,42]. Lastly, results are limited to the ∼3.5% patients hospitalized for AECOPD and may not generalize to a broader respiratory impaired population.

In conclusion, anemia affected 18.5% of hospitalized patients with AECOPD and was associated with higher postdischarge mortality and AECOPD readmission. Nutritional anemia was most common, followed by ACD. Only nutritional anemia was associated with an increased AECOPD readmission risk, whereas all subtypes were associated with higher postdischarge mortality. Subtype-specific treatment with iron, vitamin B12, folic acid, or ESA was associated with reduced postdischarge mortality risk, suggesting the benefit of targeted management.

## Author contributions

The authors’ responsibilities were as follows – VD, LL: contributed to the concept and design of the study; VD: performed the statistical analysis, interpretation, and writing under the supervision of LL; NK, LL: provided feedback to optimize the design of the study; NK, LEGWV, LL: revised the manuscript critically; and all authors: contributed to the article and approved the final version of the manuscript.

## Ethics approval

This study received approval from both the InterMutualistic Agency (IMA) and Minimal Hospital Dataset (MHD) database administrators and “Social Security and Health Chamber of the Belgian Information Security Committee” (approval code IVC/KSZG/23/008), waiving the need for individual informed consent.

## Data availability

The data that support the findings of this study are available from the InterMutualistic Agency and Minimal Hospital Dataset, but restrictions apply to the availability of these data, which were used under license for the current study, and so are not publicly available. Any further inquiries regarding data availability should be directed to Professor Lies Lahousse (lies.lahousse@ugent.be) or the administrators of the InterMutualistic Agency (IMA) database or Minimal Hospital Dataset.

## Declaration of generative AI and AI-assisted technologies in the writing process

The authors declare that no generative AI or AI-assisted technologies were used in the writing of this manuscript.

## Funding

The authors reported no funding received for this study.

## Conflict of interest

Outside this manuscript, LL has been consulted as an expert for AstraZeneca, GlaxoSmithKline, and Sanofi, and has given lectures sponsored by Chiesi, Johnson and Johnson services, IPSA vzw and Domus Medica vzw (nonprofit organizations facilitating lifelong learning for health care providers), all paid to her institution. She received support for travel from Menarini and AstraZeneca. NK is an unpaid member of the European Hematology Association, the European Myeloma Network and the Belgian Hematology Society. VD and LL are unpaid members of the European Respiratory Society and Belgian Respiratory Society. LL is a member of the Faculty Board of Ghent University—Faculty of Pharmaceutical Sciences and faculty committees. None of which is related to the content of this work. LEGWV reports payments for consultancy and/or lectures from AstraZeneca, GSK, Chiesi, Sanofi, Pulmonx, Grifols, Novartis, and Boehringer outside the scope of this manuscript. LEGWV reports research grants paid to his institution from the Family Kamprad Foundation, Svensk Lungmedicinsk Förening, the Swedish government and county council ALF, the Swedish Heart and Lung Foundation, and AstraZeneca, all outside the scope of the submitted work. All other authors declare that the research was conducted in the absence of any commercial or financial relationships that could be construed as a potential conflict of interest.

## References

[1] Fabbri L.M., Celli B.R., Agustí A., Criner G.J., Dransfield M.T., Divo M. (2023). COPD and multimorbidity: recognising and addressing a syndemic occurrence. Lancet Respir. Med..

[2] Cavaillès A., Brinchault-Rabin G., Dixmier A., Goupil F., Gut-Gobert C., Marchand-Adam S., Comorbidities of COPD (2013). Eur. Respir. Rev..

[3] Boutou A.K., Hopkinson N.S., Polkey M.I. (2015). Anaemia in chronic obstructive pulmonary disease: an insight into its prevalence and pathophysiology. Clin. Sci. (Lond)..

[4] Zhang J., DeMeo D.L., Silverman E.K., Make B.J., Wade R.C., Wells J.M. (2021). Secondary polycythemia in chronic obstructive pulmonary disease: prevalence and risk factors. BMC Pulm. Med..

[5] Sarkar M., Rajta P.N., Khatana J. (2015). Anemia in chronic obstructive pulmonary disease: prevalence, pathogenesis, and potential impact. Lung India.

[6] Similowski T., Agustí A., MacNee W., Schönhofer B. (2006). The potential impact of anaemia of chronic disease in COPD. Eur. Respir. J..

[7] Weiss G., Ganz T., Goodnough L.T. (2019). Anemia of inflammation. Blood.

[8] Pizzini A., Aichner M., Sonnweber T., Tancevski I., Weiss G., Löffler-Ragg J. (2020). The significance of iron deficiency and anemia in a real-life COPD cohort. Int. J. Med. Sci..

[9] Rahimi-Rad M.H., Sadighi T., Rabieepour M., Dinparast R., RahimiRad S. (2015). Prevalence of anemia and its impact on mortality in patients with acute exacerbation of chronic obstructive pulmonary disease in a developing country setting. Pneumologia.

[10] Vasquez A., Logomarsino J.V. (2016). Anemia in chronic obstructive pulmonary disease and the potential role of iron deficiency. COPD.

[11] Nielsen B.B.S., Springborg C.J., Jacobsen P.A., Weinreich U.M. (2025). Anemia and polycythemia in patients hospitalised with acute exacerbations of chronic obstructive pulmonary disease: prevalence, patient characteristics, and risk of readmission and mortality, Eur. Clin. Respir. J..

[12] Agusti A., Celli B.R., Criner G.J., Halpin D.M., de Oca M.M., Ozoh O. (2026). https://goldcopd.org/2026-gold-report-and-pocket-guide/.

[13] Vauterin D., Van Vaerenbergh F., Grymonprez M., Joos G., Lahousse L. (2025). Predictors of mortality and hospitalised exacerbations in obstructive airway diseases. ERJ Open Res.

[14] (2024). The Sectoral Committee of Social Security and Health, Section Health ('Informatieveiligheidscomité').

[15] IMA/AIM (2024). https://ima-aim.be/.

[16] Belgian Ministry of Health (FOD Volksgezondheid) (2024). https://www.health.belgium.be/nl/gezondheid/organisatie-van-de-gezondheidszorg/ziekenhuizen/registratiesystemen/mzg.

[17] Dehondt V., Vauterin D., Derom E., Lahousse L. (2025). Association of timely spirometry with lower all-cause mortality: a nationwide obstructive cohort study. Chest.

[18] Hansen D.L., Overgaard U.M., Pedersen L., Frederiksen H. (2016). Positive predictive value of diagnosis coding for hemolytic anemias in the Danish National Patient Register. Clin. Epidemiol..

[19] Zalfani J., Frøslev T., Olsen M., Ben Ghezala I., Gammelager H., Arendt J.F. (2012). Positive predictive value of the International Classification of Diseases, 10th edition diagnosis codes for anemia caused by bleeding in the Danish National Registry of Patients. Clin. Epidemiol..

[20] Barcellini W., Fattizzo B. (2020). The changing landscape of autoimmune hemolytic anemia. Front Immunol.

[21] Madu A.J., Ughasoro M.D. (2017). Anaemia of chronic disease: an in-depth review, Med. Princ. Pract..

[22] Segal J.B., Chang H.Y., Du Y., Walston J.D., Carlson M.C., Varadhan R. (2017). Development of a claims-based frailty indicator anchored to a well-established frailty phenotype, Med. Care.

[23] Sonani H., Dhaduk K., Dankhara N., Viroliya K., Desai C.H., Sonani A.A. (2023). Anemia as a significant predictor of adverse outcomes in hospitalized patients with acute exacerbations of chronic obstructive pulmonary disease: analysis of national (Nationwide) inpatient sample database. Cureus.

[24] Wu S., Yi Q., Luo Y., Wei H., Ge H., Liu H. (2025). Hemoglobin and clinical outcomes of in-hospital patients with severe acute exacerbation of chronic obstructive pulmonary disease: a multicenter cohort study. Front. Med. (Lausanne)..

[25] Park S.C., Kim Y.S., Kang Y.A., Park E.C., Shin C.S., Kim D.W. (2018). Hemoglobin and mortality in patients with COPD: a nationwide population-based cohort study. Int. J. Chron. Obstruct. Pulmon. Dis..

[26] Barba R., de Casasola G.G., Marco J., Emilio Losa J., Plaza S., Canora J. (2012). Anemia in chronic obstructive pulmonary disease: a readmission prognosis factor. Curr. Med. Res. Opin..

[27] Balasubramanian A., Henderson R.J., Putcha N., Fawzy A., Raju S., Hansel N.N. (2021). Haemoglobin as a biomarker for clinical outcomes in chronic obstructive pulmonary disease. ERJ Open Res.

[28] Takizawa A., Shimada T., Chubachi S., Arai T., Miyakawa A., Iizuka H. (2025). Exploring the pathophysiology of anemia in COPD: insights from chest CT and longitudinal clinical data. Respir. Med..

[29] Hsieh M.J., Yeh J.K., Huang Y.C., Ho M.Y., Chen D.Y., Lee C.H. (2024). Cardiac power output associated with hospitalization and mortality in coronary artery disease patients at stage B heart failure. Int. J. Cardiol. Heart Vasc.

[30] Movahed M.R., Ramaraj R., Manrique C., Hashemzadeh M. (2022). Left ventricular hypertrophy is independently associated with all-cause mortality. Am. J. Cardiovasc. Dis..

[31] Müllerová H., Marshall J., de Nigris E., Varghese P., Pooley N., Embleton N. (2022). Association of COPD exacerbations and acute cardiovascular events: a systematic review and meta-analysis. Ther. Adv. Respir. Dis..

[32] Silverberg D.S., Mor R., Weu M.T., Schwartz D., Schwartz I.F., Chernin G. (2014). Anemia and iron deficiency in COPD patients: prevalence and the effects of correction of the anemia with erythropoiesis stimulating agents and intravenous iron. BMC Pulm. Med..

[33] Amado C.A., Ghadban C., Agüero J., Lavín B.A., Martín-Audera P., Guerra A.R. (2025). Non-anemic iron deficiency predicts COPD exacerbations and hospitalizations: results from a prospective cohort. J. Clin. Med..

[34] Barnes P.J. (2022). Oxidative stress in chronic obstructive pulmonary disease. Antioxidants (Basel).

[35] Vogelmeier C.F., Friedrich F.W., Timpel P., Kossack N., Diesing J., Pignot M. (2025). Comorbidities and cause of death in COPD patients compared to Non-COPD controls: an 8-year observational retrospective healthcare claims database cohort study. Int. J. Chron. Obstruct. Pulmon. Dis..

[36] Alisamir M., Ebrahimi M., Rahim F. (2022). Anemia in chronic obstructive pulmonary disease: a systematic review. Respir. Investig..

[37] Pérez-Peiró M., Martín-Ontiyuelo C., Rodó-Pi A., Piccari L., Admetlló M., Durán X. (2021). Iron replacement and redox balance in non-anemic and mildly anemic iron deficiency COPD patients: insights from a clinical trial. Biomedicines.

[38] Nguyen H.Q., Chu L., Amy Liu I.L., Lee J.S., Suh D., Korotzer B. (2014). Associations between physical activity and 30-day readmission risk in chronic obstructive pulmonary disease. Ann. Am. Thorac. Soc..

[39] Cireli E., Mertoğlu A. (2022). The impact of anemia on the mortality of COPD patients hospitalized for acute exacerbation resulting in respiratory failure. Monaldi Arch, Chest Dis.

[40] Poggiali E., Migone De Amicis M., Motta I. (2014). Anemia of chronic disease: a unique defect of iron recycling for many different chronic diseases. Eur. J. Intern. Med..

[41] Mizumura K., Gon Y. (2021). Iron-regulated reactive oxygen species production and programmed cell death in chronic obstructive pulmonary disease. Antioxidants (Basel).

[42] Ho T., Nichols M., Nair G., Radford K., Kjarsgaard M., Huang C. (2022). Iron in airway macrophages and infective exacerbations of chronic obstructive pulmonary disease. Respir. Res..

