# Supplementary material for: Nutritional anemia is associated with increased risk of severe COPD exacerbations: a prospective cohort study
Source: Am J Clin Nutr. 2026 Jun 20;124(2):101402. doi: 10.1016/j.ajcnut.2026.101402 (PMC13494100; doi:10.1016/j.ajcnut.2026.101402)
Supplement: Supplementary file 1 [file mmc1.docx]

Supplementary Materials

Nutritional anemia is associated with increased risk of severe COPD exacerbations: a prospective cohort study

Valerie Dehondt

Table of Contents

[Supplement Methods	4](#_Toc230853471)

[Supplemental tables	5](#_Toc230853472)

[eTable 1: STROBE reporting guideline	5](#_Toc230853473)

[eTable 2: Definition of in- and exclusion criteria, demographics, exposure, comorbidities, disease severity-related variables, treatment, clinical risk scores and outcomes	7](#_Toc230853474)

[eTable 3: Cox proportional hazards models of post-discharge mortality using inverse probability of treatment weighting for iron therapy, including interaction terms with anemia subtypes	17](#_Toc230853475)

[eTable 4: Cox proportional hazards models of post-discharge mortality using inverse probability of treatment weighting for vitamin B12 therapy, including interaction terms with anemia subtypes	18](#_Toc230853476)

[eTable 5: Cox proportional hazards models of post-discharge mortality using inverse probability of treatment weighting for folic acid therapy, including interaction terms with anemia subtypes	19](#_Toc230853477)

[eTable 6: Cox proportional hazards models of post-discharge mortality using inverse probability of treatment weighting for ESA therapy, including interaction terms with anemia subtypes	20](#_Toc230853478)

[Supplemental Figures	21](#_Toc230853479)

[eFigure 1: Overview of the study design	21](#_Toc230853480)

[eFigure 2: Flowchart of study population.	22](#_Toc230853481)

[eFigure 3: Love plots	23](#_Toc230853482)

[eFigure 4: Kaplan-Meier curve comparing patients with and without anemia	27](#_Toc230853483)

[eFigure 5: Logistic regression analyses for the association between anemia and in-hospital mortality in COPD patients hospitalized for an exacerbation	28](#_Toc230853484)

[eFigure 6 sensitivity analyses: Cox analyses for the association between anemia and post-discharge mortality adjusted for comorbidities	29](#_Toc230853485)

[eFigure 7 sensitivity analyses: Cox analyses for the association between anemia subtypes and post-discharge mortality in COPD patients hospitalized for an exacerbation (without reclassification of other/unspecified anemia)	30](#_Toc230853486)

[eFigure 8 sensitivity analyses: Cox analyses for the association between anemia subtypes and readmission for severe exacerbation in COPD patients hospitalized for an exacerbation (without reclassification of other/unspecified anemia)	31](#_Toc230853487)

[eFigure 9 sensitivity analyses: Logistic regression analyses for the association between anemia and in-hospital mortality in COPD patients hospitalized for an exacerbation (without reclassification of other/unspecified anemia)	32](#_Toc230853488)

[eFigure 10 sensitivity analyses: Cox analyses for the association between anemia subtypes and post-discharge mortality in COPD patients hospitalized for an exacerbation, without reclassification and excluding other/unspecified anemia	33](#_Toc230853489)

[eFigure 11 sensitivity analyses: Cox analyses for the association between anemia subtypes and readmission for severe exacerbation in COPD patients hospitalized for an exacerbation, without reclassification and excluding other/unspecified anemia	34](#_Toc230853490)

[eFigure 12 sensitivity analyses: Logistic regression analyses for the association between anemia and in-hospital mortality in COPD patients hospitalized for an exacerbation, without reclassification and excluding other/unspecified anemia	35](#_Toc230853491)

[eFigure 13 sensitivity analyses: Cox analyses for the association between anemia subtypes and readmission for severe exacerbation in COPD patients hospitalized for an exacerbation using Fine-Gray subdistribution hazard model (Model 2)	36](#_Toc230853492)

[References	37](#_Toc230853493)

# Supplement Methods

The source population was provided by the InterMutualistic Agency (IMA) and Minimal Hospital Dataset (MHD). The IMA centralizes all claims data from Belgian health insurance funds and manages three databases: (1) a population database containing socio-demographic characteristics (e.g. age and sex), (2) a healthcare database containing data on reimbursed ambulatory and hospital care (e.g. medical procedures, inpatient medication and other reimbursed care) and (3) a pharmaceutical database containing outpatient medication prescription claims (e.g., dispensing date, Anatomical Therapeutic Chemical [ATC] Classification code). Data are available for all legal residents who are affiliated with a Belgian health insurance fund.(1,2) MHD, collected by the Belgian Ministry of Health, collects discharge diagnoses for each hospital admission, coded in International Classification of Diseases (ICD) codes (ICD-10).(3) Data from IMA and MHD were linked by a Trusted Third Party (eHealth).(1)

# Supplemental tables

## eTable 1: STROBE reporting guideline

|  | **Item No.** | **Recommendation** | **Page No.** |
| --- | --- | --- | --- |
| **Title and abstract** | 1 | (*a*) Indicate the study’s design with a commonly used term in the title or the abstract | 1, 4 |
|  |  | (*b*) Provide in the abstract an informative and balanced summary of what was done and what was found | 4-5 |
| **Introduction** | | |  |
| Background/rationale | 2 | Explain the scientific background and rationale for the investigation being reported | 6 |
| Objectives | 3 | State specific objectives, including any prespecified hypotheses | 6-7 |
| **Methods** | | |  |
| Study design | 4 | Present key elements of study design early in the paper | 7 |
| Setting | 5 | Describe the setting, locations, and relevant dates, including periods of recruitment, exposure, follow-up, and data collection | 7-8, Supplement Methods, eFigure 1 |
| Participants | 6 | (*a*) Give the eligibility criteria, and the sources and methods of selection of participants. Describe methods of follow-up | 7, eFigure 1&2 |
| Variables | 7 | Clearly define all outcomes, exposures, predictors, potential confounders, and effect modifiers. Give diagnostic criteria, if applicable | 8-9, eTable 2 |
| Data sources/ measurement | 8* | For each variable of interest, give sources of data and details of methods of assessment (measurement). Describe comparability of assessment methods if there is more than one group | 8, eTable 2 |
| Bias | 9 | Describe any efforts to address potential sources of bias | 9-11 |
| Study size | 10 | Explain how the study size was arrived at | 8, Supplement Methods, eFigure 2 |
| Quantitative variables | 11 | Explain how quantitative variables were handled in the analyses. If applicable, describe which groupings were chosen and why | 8-9 |
| Statistical methods | 12 | (*a*) Describe all statistical methods, including those used to control for confounding | 9-11 |
|  |  | (*b*) Describe any methods used to examine subgroups and interactions | 11 |
|  |  | (*c*) Explain how missing data were addressed | 7-8 |
|  |  | (*d*) If applicable, explain how loss to follow-up was addressed | NA |
|  |  | (*e*) Describe any sensitivity analyses | 11 |
| **Results** | | |  |
| Participants | 13* | (a) Report numbers of individuals at each stage of study—eg numbers potentially eligible, examined for eligibility, confirmed eligible, included in the study, completing follow-up, and analysed | 11-12,  Table 1, eFigure 2 |
|  |  | (b) Give reasons for non-participation at each stage | NA |
|  |  | (c) Consider use of a flow diagram | eFigure 2 |
| Descriptive data | 14* | (a) Give characteristics of study participants (eg demographic, clinical, social) and information on exposures and potential confounders | Table 1 |
|  |  | (b) Indicate number of participants with missing data for each variable of interest | 7-8,  eFigure 2 |
|  |  | (c) Summarise follow-up time (eg, average and total amount) | 12 |
| Outcome data | 15* | Report numbers of outcome events or summary measures over time | 11-13 |
| Main results | 16 | (*a*) Give unadjusted estimates and, if applicable, confounder-adjusted estimates and their precision (eg, 95% confidence interval). Make clear which confounders were adjusted for and why they were included | 12-14,  Figure 1&3 |
|  |  | (*b*) Report category boundaries when continuous variables were categorized | NA |
|  |  | (*c*) If relevant, consider translating estimates of relative risk into absolute risk for a meaningful time period | NA |
| Other analyses | 17 | Report other analyses done—eg analyses of subgroups and interactions, and sensitivity analyses | 14 |
| **Discussion** | | |  |
| Key results | 18 | Summarise key results with reference to study objectives | 14 |
| Limitations | 19 | Discuss limitations of the study, taking into account sources of potential bias or imprecision. Discuss both direction and magnitude of any potential bias | 17-18 |
| Interpretation | 20 | Give a cautious overall interpretation of results considering objectives, limitations, multiplicity of analyses, results from similar studies, and other relevant evidence | 14-17 |
| Generalisability | 21 | Discuss the generalisability (external validity) of the study results | 14-18 |
| **Other information** | | |  |
| Funding | 22 | Give the source of funding and the role of the funders for the present study and, if applicable, for the original study on which the present article is based | 1 |

## eTable 2: Definition of in- and exclusion criteria, demographics, exposure, comorbidities, disease severity-related variables, treatment, clinical risk scores and outcomes

| **VARIABLE** | | **ICD, ATC AND MEDICAL PROCEDURE CODES** |
| --- | --- | --- |
| **INCLUSION CRITERIA(4,5)** | | |
| Drugs for obstructive airway diseases | | **ATC:** R03  Short-acting bronchodilators ~ ATC: R03AC02, R03AC03, R03AL01, R03AL02, R03BB01  Long-acting bronchodilators ~ ATC: R03AC12, R03AC13, R03AC18, R03AC19, R03AL03-R03AL06, R03BB04-R03BB07  Inhaled corticosteroids ~ ATC: R03BA01, R03BA02, R03BA05  Combination therapy of long-acting β2-agonists and inhaled corticosteroids ~ due to use of the ATC for the specific categories or use of ATC: R03AK06 – R03AK08, R03AK10 – R03AK12, R03AK14  Multiple R03 medication: R03AL08, R03AL09, R03AL11, R03AL12, R03BC01, R03CC02, R03CC03, R03DA01, R03DA04, R03DC01, R03DC03, R03DC53, R03DX05, R03DX08-R03DX10 |
| ≥45 years | | Age ≥45 years on index date |
| ≥1 year coverage | | ≥1 year coverage by a Belgian health insurance fund |
| Hospitalized COPD exacerbation | | Hospital admissions or emergency department visits with a primary diagnosis code of (acute) COPD exacerbation, COPD with acute lower respiratory infection (**ICD-10:** J44.0 or J44.1)  OR  with a primary diagnosis of chronic lower respiratory disease (**ICD-10:** J40-J47) or respiratory failure (**ICD-10:** J96), combined with a secondary diagnosis code of (acute) COPD exacerbation, COPD with acute lower respiratory infection (**ICD-10:** J44.0 or J44.1).  Hospitalizations with a concomitant pneumonia diagnosis were excluded (**ICD-10:** A01.03, A02.22, A37.01, A37.11, A37.81, A37.91, A50.04, A54.84, B01.2, B05.2, B06.81, B77.81, J09.X1, J09.X2, J09.X3, J10.0, J11.0, J12, J13, J14, J15, J16, J17, J18, J84.11, J84.2, J85.1, J95.851). |
| **DEMOGRAFICS (on index date)(4-6)** | | |
| Age | | Age on index date based on the year and month of birth, not the exact date due to patient privacy. |
| Sex | | Sex on index date |
| (Ever-)Smoker | | **ICD-9:** 305.1, V15.82  **ICD-10:** F17, Z71.6, Z72.0, Z87.891  **ATC**: N07BA  **Medical procedure code:** 740434, 740445, 740456, 740460, 740471, 740482 |
| **EXPOSURE** | | |
| Chronic anemia | | **ICD-10:** D46.0-D46.4, D50-D53, D55-D61, D63-D64 |
|  | Hematologic anemia | **ICD-10:**  Haemolytic anemia: D55-D59  Aplastic anemia: D61, D64.81  Refractory/myelodysplastic anemia: D46.0-D46.4, D64.0-D64.2  Or unspecified/other anemia (**ICD-10**: D64.89, D64.9) + diagnosis of specific blood cancer or myelodysplastic syndrome (definition mentioned below: ‘Non-hodgkin lymphoma’, ‘Waldenström’, ‘Leukemia’, ‘Multiple myeloma’, ‘Myelodysplastic syndrome’) |
|  | Anemia of chronic disease | **ICD-10:** D63  Or unspecified/other anemia (**ICD-10**: D64.89, D64.9) + diagnosis of other cancers, HIV, respiratory tuberculosis, autoimmune diseases (rheumatoid arthritis, systemic lupus erythematosus, connective-tissue diseases, vasculitis, sarcoidosis, inflammatory bowel disease), chronic rejection after solid-organ transplantation, or chronic kidney disease (definition mentioned below: ‘Other cancer’, ‘HIV’, ‘Respiratory tuberculosis’, ‘Rheumatoid arthritis’, ‘Systemic lupus erythematosus’, ‘Connective tissue disease’, ‘Vasculitis’, ‘Sarcoidosis’, ‘Inflammatory bowel disease’ , ‘Chronic rejection after solid-organ transplantation’, ‘Chronic kidney disease’) |
|  | Nutritional anemia | **ICD-10:** D50-D53  Or unspecified/other anemia (**ICD-10**: D64.89, D64.9) + diagnosis of malnutrition (definition mentioned below: ‘Malnutrition’) |
|  | Other types of anemia | **ICD-10:** D64.89, D64.9 without any of additional diagnoses of above |
| **COMORBIDITIES (≤1 year before index date)**(4-6) | | |
| Respiratory tuberculosis | | **ICD-10:** A15, B90.9  **Medical procedure code:** 549614, 549625, 549636, 549640 |
| Non-hodgkin lymphoma | | **ICD-10:** C82-C86  **Medical procedure codes:** 588453, 588464, 588475, 588486 |
| Waldenström | | **ICD-10:** C88.0  **Medical procedure codes:** 535931, 535942, 594613, 594624 |
| Leukemia | | **ICD-10:** C90.1, C91-C95, D47.5  **Medical procedure codes:** 535570, 535581, 535592, 535603, 535614, 535625, 535710, 535721, 535754, 535765, 535791, 535802, 535813, 535824, 535835, 535846, 535850, 535861, 535975, 535986, 535990, 536001, 587834, 587845, 587871, 587882, 587893, 587904, 588431, 588442, 588490, 588501, 588593, 588604, 594495, 594506, 594510, 594521, 594532, 594543, 594554, 594565, 594576, 594580, 594591, 594602, 594635, 594646, 594753, 594764, 594775, 594786, 594790, 594801, 594812, 594823, 594834, 594845, 594856, 594860, 594871, 594882, 594893, 594904, 594915, 594926, 594930, 594941, 595033, 595044, 595055, 595066, 595254, 595265 |
| Multiple myeloma | | **ICD-10:** C90.0  **Medical procedure codes:** 587871, 587882, 588453, 588464 |
| Myelodysplastic syndrome | | **ICD-10:** D46  **Medical procedure codes:** 535636, 535640, 535651, 535662, 535695, 535706 |
| Other cancer | | **ICD-10:** C00-C81, C87, C88.1-C88.9, C90.2-C90.3, C96, Z51.0, Z51.11, Z51.12  **ATC:** L01  **Medical procedure code:** 154873, 154884, 154895, 154906, 157231, 157242, 201191, 201202, 201213, 201224, 220275, 220286, 220371, 220382, 226914, 226925, 226936, 226940, 227216, 227220, 227275, 227286, 227636, 227640, 227651, 227662, 227673, 227684, 227695, 227706, 227710, 227721, 227732, 227743, 227754, 227765, 227776, 227780, 227791, 227802, 227813, 227824, 227835, 227846, 230473, 230484, 231033, 231044, 235152, 235163, 241231, 241242, 241415, 241426, 241430, 241441, 241452, 241463, 242012, 242023, 242034, 242045, 242292, 242303, 242314, 242325, 242830, 242841, 242852, 242863, 242874, 242885, 242896, 242900, 243051, 243062, 243073, 243084, 243235, 243246, 243736, 243740, 243751, 243762, 243773, 243784, 244016, 244020, 244031, 244042, 244075, 244086, 244311, 244322, 244856, 244860, 244893, 244904, 244915, 244926, 244930, 244941, 244952, 244963, 244974, 244985, 245512, 245523, 245534, 245545, 246050, 246061, 246072, 246083, 247111, 247122, 247133, 247144, 251753, 251764, 251775, 251786, 254892, 254903, 256115, 256126, 256336, 256340, 256572, 256583, 256771, 256782, 257191, 257202, 258355, 258366, 258370, 258381, 258392, 258403, 258451, 258462, 258554, 258565, 258856, 258860, 258871, 258882, 258893, 258904, 259033, 259044, 259114, 259125, 260190, 260201, 260411, 260422, 260433, 260444, 260551, 260562, 260654, 260665, 260750, 260761, 261111, 261122, 261391, 261402, 261472, 261483, 261671, 261682, 261774, 261785, 261796, 261800, 262334, 262345, 262570, 262581, 277756, 277760, 277771, 277782, 278795, 278806, 278810, 278821, 280136, 280140, 280151, 280162, 281831, 281842, 281956, 281960, 282310, 282321, 282671, 282682, 288455, 288466, 288470, 288481, 310494, 310505, 311312, 311323, 312550, 312561, 312572, 312583, 312594, 312605, 312653, 312664, 312970, 312981, 317111, 317122, 350070, 350092, 350114, 350125, 350136, 350140, 350232, 350254, 350265, 350276, 350280, 350291, 350302, 350372, 350383, 350394, 350405, 350416, 350420, 350674, 350685, 350696, 350700, 431174, 431185, 433016, 433020, 435831, 435842, 436295, 436306, 436376, 436380, 444113, 444124, 444135, 444146, 444150, 444161, 444172, 444183, 444194, 444205, 444216, 444220, 444231, 444242, 444253, 444264, 444275, 444286, 444290, 444301, 444312, 444323, 444334, 444345, 444356, 444360, 444371, 444382, 444393, 444404, 444415, 444426, 444430, 444441, 444452, 444463, 444474, 444485, 444496, 444500, 444511, 444522, 444533, 444544, 444555, 444566, 444570, 444581, 444592, 444603, 444636, 444640, 444651, 444662, 444673, 444684, 473970, 473981, 474795, 474806, 532696, 532700, 532711, 532722, 548575, 548586, 565073, 565084, 565095, 565106, 565110, 565121, 565132, 565143, 565154, 565165, 587915, 587926, 588442, 588512, 588523, 588534, 588545, 588556, 588560, 588571, 588582, 588770, 588781, 588976, 588980, 589691, 589702, 589713, 589724, 589831, 589842, 589875, 589886, 594252, 594263, 594274, 594285, 594296, 594300, 594311, 594322, 594333, 594344, 594355, 594366, 594370, 594381, 594392, 594403, 594414, 594425, 594436, 594440, 594451, 594462, 594694, 594705, 594716, 594720, 597273, 597295, 598581, 745010, 745021, 745032, 745043, 745113, 745124, 745135, 745146, 745150, 745161, 771632, 771643 |
| Any malignancy including non-hodgkin lymphoma, waldenström, leukemia and multiple myeloma | | **ICD-10:** C00-C76, C80.1, C80.2, C81-C96, Z51.0, Z51.11, Z51.12  **ATC:** L01  **Medical procedure code:** 154873, 154884, 154895, 154906, 157231, 157242, 201191, 201202, 201213, 201224, 220275, 220286, 220371, 220382, 226914, 226925, 226936, 226940, 227216, 227220, 227275, 227286, 227636, 227640, 227651, 227662, 227673, 227684, 227695, 227706, 227710, 227721, 227732, 227743, 227754, 227765, 227776, 227780, 227791, 227802, 227813, 227824, 227835, 227846, 230473, 230484, 231033, 231044, 235152, 235163, 241231, 241242, 241415, 241426, 241430, 241441, 241452, 241463, 242012, 242023, 242034, 242045, 242292, 242303, 242314, 242325, 242830, 242841, 242852, 242863, 242874, 242885, 242896, 242900, 243051, 243062, 243073, 243084, 243235, 243246, 243736, 243740, 243751, 243762, 243773, 243784, 244016, 244020, 244031, 244042, 244075, 244086, 244311, 244322, 244856, 244860, 244893, 244904, 244915, 244926, 244930, 244941, 244952, 244963, 244974, 244985, 245512, 245523, 245534, 245545, 246050, 246061, 246072, 246083, 247111, 247122, 247133, 247144, 251753, 251764, 251775, 251786, 254892, 254903, 256115, 256126, 256336, 256340, 256572, 256583, 256771, 256782, 257191, 257202, 258355, 258366, 258370, 258381, 258392, 258403, 258451, 258462, 258554, 258565, 258856, 258860, 258871, 258882, 258893, 258904, 259033, 259044, 259114, 259125, 260190, 260201, 260411, 260422, 260433, 260444, 260551, 260562, 260654, 260665, 260750, 260761, 261111, 261122, 261391, 261402, 261472, 261483, 261671, 261682, 261774, 261785, 261796, 261800, 262334, 262345, 262570, 262581, 277756, 277760, 277771, 277782, 278795, 278806, 278810, 278821, 280136, 280140, 280151, 280162, 281831, 281842, 281956, 281960, 282310, 282321, 282671, 282682, 288455, 288466, 288470, 288481, 310494, 310505, 311312, 311323, 312550, 312561, 312572, 312583, 312594, 312605, 312653, 312664, 312970, 312981, 317111, 317122, 350070, 350092, 350114, 350125, 350136, 350140, 350232, 350254, 350265, 350276, 350280, 350291, 350302, 350372, 350383, 350394, 350405, 350416, 350420, 350674, 350685, 350696, 350700, 431174, 431185, 433016, 433020, 435831, 435842, 436295, 436306, 436376, 436380, 444113, 444124, 444135, 444146, 444150, 444161, 444172, 444183, 444194, 444205, 444216, 444220, 444231, 444242, 444253, 444264, 444275, 444286, 444290, 444301, 444312, 444323, 444334, 444345, 444356, 444360, 444371, 444382, 444393, 444404, 444415, 444426, 444430, 444441, 444452, 444463, 444474, 444485, 444496, 444500, 444511, 444522, 444533, 444544, 444555, 444566, 444570, 444581, 444592, 444603, 444636, 444640, 444651, 444662, 444673, 444684, 473970, 473981, 474795, 474806, 532696, 532700, 532711, 532722, 548575, 548586, 565073, 565084, 565095, 565106, 565110, 565121, 565132, 565143, 565154, 565165, 587834, 587845, 587871, 587882, 587893, 587904, 587915, 587926, 588431, 588442, 588453, 588464, 588475, 588486, 588490, 588501, 588512, 588523, 588534, 588545, 588556, 588560, 588571, 588582, 588593, 588604, 588770, 588781, 588976, 588980, 589691, 589702, 589713, 589724, 589831, 589842, 589875, 589886, 594252, 594263, 594274, 594285, 594296, 594300, 594311, 594322, 594333, 594344, 594355, 594366, 594370, 594381, 594392, 594403, 594414, 594425, 594436, 594440, 594451, 594462, 594495, 594506, 594510, 594521, 594532, 594543, 594554, 594565, 594576, 594580, 594591, 594602, 594613, 594624, 594635, 594646, 594694, 594705, 594716, 594720, 594753, 594764, 594775, 594786, 594790, 594801, 594812, 594823, 594834, 594845, 594856, 594860, 594871, 594882, 594893, 594904, 594915, 594926, 594930, 594941, 597273, 597295, 598581, 745010, 745021, 745032, 745043, 745113, 745124, 745135, 745146, 745150, 745161, 771632, 771643 |
| Metastatic cancer | | **ICD-10:** C77-C79, C80.0 |
| Hypertension | | **ICD-10:** I10, I11, I12, I13, I15, I16, I67.4  **ATC: combination of treatment of ≥2 of the following drug classes(7)**:   I) **Cardioselective beta blocker:** C07AB, C07FX03, C07FX04, C07FX05, C07FX06 II) **ACE inhibitor or angiotensin II receptor blocker:** C09A, C09BA, C09C, C09DA, C09DX02, C09DX04, C10BX04, C10BX06, C10BX10, C10BX12, C10BX15, C10BX16, C10BX17, C10BX20, C10BX21 III) **Calcium channel blocker:** C08C, C09XA53, C10BX03, C10BX09 IV) **Non-loop diuretic:** C03A, C03BA, C03BB, C03EA, C07BA, C07BG, C07DA, C09XA52 V) **Other antihypertensive drugs (alpha adrenergic blocker, vasodilator):** C02A, C02B, C02C, C02DB, C02DD, C02DG, C02L   **or use of ≥1 combination product**: C07BB, C07CB, C07DB, C07FB, C08G, C09BB, C09BX, C09DB, C09DX01, C09DX03, C09DX05, C09DX06, C09DX07, C09DX08, C09XA54, C10BX07, C10BX11, C10BX13, C10BX14, C10BX18, C10BX19 |
| Myocardial infarction | | **ICD-10:** I21, I22, I25.2 |
| Cerebrovascular disease | | **ICD-10:** G45, G46, H34.0, I60, I61, I62, I63, I65, I66, I67, I68, I69, Z86.73  **Medical procedure code:** 182136, 182140, 182151, 182162, 182173, 182184, 477724, 477746, 477761, 477783 |
| Stroke | | **ICD-10:** I61, I62, I63, I67.89, I69.1, I69.2, I69.3, I69.8, I69.9, I97.82, Z86.73  **Medical procedure code:** 182136, 182140, 182151, 182162, 182173, 182184, 477724, 477746, 477761, 477783 |
| Heart failure | | **ICD-10:** I09.81, I11.0, I13.0, I13.2, I25.5, I42.0, I42.6-I42.9, I43, I50, P29.0  **ATC: combination of treatment of all of the following drug classes(7):**   I) **Beta blocker (selective or alpha and beta blocking):** C07AB, C07AG, C07BB, C07BG, C07CB, C07CG, C07DB, C07FB, C07FX03, C07FX04, C07FX05, C07FX06 II) **ACE inhibitor or angiotensin II receptor blocker:** C09A, C09BA, C09BB, C09C, C09DA, C09DB, C09DX01, C09DX02, C09DX03, C09DX04, C09DX06, C09DX07, C09DX08, C10BX04, C10BX06, C10BX07, C10BX10, C10BX11, C10BX12, C10BX13, C10BX14, C10BX15, C10BX16, C10BX17, C10BX18, C10BX19, C10BX20, C10BX21 III) **Loop diuretic:** C03C, C03EB   **or use of ≥1 combination product (beta blocker + ACE inhibitor or beta blocker + angiotensin II receptor blocker)**: C09BX02, C09BX04, C09BX05, C09BX06, C09BX07, C09DX05 **and loop diuretic** |
| Arrythmia | | **ICD-10:** I44, I45, I47, I48, I49  **ATC:** C01EB10, C01B, C07AA07, C07BA07, C07FX02,  **Medical procedure code:** 180272, 180283, 180294, 180305, 589514, 589525, 589551, 589562 |
| Peripheral vascular disease | | **ICD-10:** I70, I71, I73.1, I73.8, I73.9, I74, I77, I79.0, K55.1, K55.8, K55.9, Z95.82, Z98.62  **Medical procedure code:** 229294, 229305, 229316, 229320, 229331, 229342, 235071, 235082, 235093, 235104, 235115, 235126, 235196, 235200, 235211, 235222, 236014, 236025, 236036, 236040, 236051, 236062, 237016, 237020, 237031, 237042, 237053, 237064, 237075, 237086, 237090, 237101, 237171, 237182, 589050, 589061, 589094, 589105, 589175, 589186, 589595, 589606, 589610, 589621, 589632, 589643, 589654, 589665 |
| Diabetes mellitus  (DM) | | **ICD-10:** E08, E09, E10, E11, E13, E14, Z46.81, Z96.41  **ATC**: A10  **Medical procedure code:** 102852, 107015, 107030, 107052, 107074, 109594, 174370, 174381, 174392, 174403, 174414, 174425, 174436, 174440, 174451, 174462, 174473, 174484, 174495, 174506, 174510, 174521, 423135, 423150, 423172, 423194, 423216, 423231, 423334, 423813, 423835, 423850, 433554, 433565, 540772, 540783, 543712, 543723, 653671, 653682, 697093, 697104, 754176, 754191, 754250, 754272, 754736, 757352, 757374, 757396, 757411, 757514, 757536, 757551, 757573, 770033, 770055, 770070, 771573, 771595, 773393, 773496, 784630, 784641, 784652, 784663, 785735, 785750, 785772, 785794, 785816, 785831, 785853, 785875, 785890, 785912, 785934, 785956, 786015, 786030, 786100, 788756, 788771, 788793, 788815, 788830, 788852, 788874, 788896, 788911, 788933, 788955, 789751, 789773, 789795, 789810, 789832, 789854, 789876, 789891, 789913, 789935, 794032, 794054, 794076, 794091, 794253, 794275, 794290, 794312, 794334, 794356, 794371, 794393, 794415, 794430, 794452, 961295, 961306, 961332, 961343 |
| Without vascular complications | | **ICD-10:** E08.0, E08.1, E08.6, E08.9, E09.0, E09.1, E09.6, E09.9, E10.1, E10.6, E10.9, E11.0, E11.1, E11.6, E11.9, E13.0, E13.1, E13.6, E13.9 |
| With vascular complications | | **ICD-10:** E08.2, E08.3, E08.4, E08.5, E08.8, E09.2, E09.3, E09.4, E09.5, E09.8, E10.2, E10.3, E10.4, E10.5, E10.8, E11.2, E11.3, E11.4, E11.5, E11.8, E13.2, E13.3, E13.4, E13.5, E13.8  **Medical procedure code:** 653671, 653682, 697093, 697104, 770070, 773393, 773496 |
| Obesity and overweight(8) | | **ICD-10:** E66, Z68.25-Z68.29, Z68.3, Z68.4  **ATC:** A08AA62, A08AB01  **Medical procedure code:** 241776, 241780, 241813, 241824, 241835, 241846 |
| Cachexia and underweight | | **ICD-10:** R64, Z68.1 |
| Peptic ulcer disease | | **ICD-10**: K22.1, K25, K26, K27, K28, Z87.11  **ATC:** A02BD04, A02BD08, A02BD11  **Medical procedure code:** 550093, 550104, 552370, 552381 |
| Chronic kidney disease | | **ICD-10:** I12.0, I13.11, I13.2, N03.2, N03.3, N03.4, N03.5, N03.6, N03.7, N05.2, N05.3, N05.4, N05.5, N05.6, N05.7, N18, N19, N25.0, T86.1, Z48.22, Z49, Z91.15, Z94.0, Z99.2  **Medical procedure code:** 107096, 107111, 107133, 107155, 318010, 318021, 318290, 318301, 470293, 470304, 470315, 470326, 470330, 470341, 470352, 470374, 470385, 470400, 470422, 470433, 470444, 470466, 470470, 470481, 470875, 470890, 470901, 470912, 470934, 470945, 471111, 471122, 471133, 471144, 471155, 471166, 471170, 471181, 474714, 474725, 754294, 757433, 757492, 761272, 761283, 761456, 761471, 761493, 761515, 761526, 761530, 761552, 761574, 761596, 761655, 761670, 767594, 767616, 767631, 767664, 767686, 767701, 767723, 767734, 767756, 767782, 767804, 767815, 767826, 767830, 767841, 767955, 767966 |
| Urinary tract infection(9) | | **ICD-10:** A56.01, N10, N12, N13.6, N15.1, N15.9, N16, N28.84, N28.85, N28.86, N30.0, N30.8, N30.9, N34, N39.0  **ATC:** J01XE01, J01XX01 |
| Mild liver disease | | **ICD-10:** B17.0, B17.10, B18, B19.10, B19.20, K70.0, K70.1, K70.2, K70.3, K70.9, K71.3, K71.4, K71.5, K71.6, K71.7, K71.8, K71.9, K73, K74, K75.2, K75.3, K75.4, K75.8, K75.9, K76.0, K76.2, K76.3, K76.4, K76.89, K76.9, Z94.4  **ATC:** J05AF07, J05AF08, J05AF10, J05AP  **Medical procedure code:** 318076, 318080, 318334, 318345, 472113, 472124, 556754, 556765, 556776, 556780, 589352, 589363 |
| Moderate and severe liver disease | | **ICD-10:** B15.0, B16.0, B16.2, B17.11, B19.0, B19.11, B19.21, I85, I86.4, K70.4, K71.1, K72.1, K72.9, K76.5, K76.6, K76.7 |
| Hemiplegia/paraplegia | | **ICD-10:** G04.1, G11.4, G80.0, G80.1, G80.2, G81, G82, G83.0, G83.4, G83.9  **Medical procedure code:** 643414, 643425 |
| History of falling | | **ICD-10:** R29.6, V00.141, V00.811, V00.831, V81.5, V81.6, V82.5, V82.6, V92.0, V93.3, V94.0, V97.0, W00, W01, W03, W05-W15, W16.0-W16.4, W17, W18, W19, Y21.1, Y21.3, Y30, Z91.81 |
| Impaired mobility | | **ICD-10:** Z74.01, Z74.09, Z99.3  **Medical procedure code:** 520015, 520026, 520030, 520041, 520052, 520063, 520074, 520085, 520096, 520100, 520111, 520122, 520133, 520144, 520155, 520166, 520170, 520181, 520192, 520203, 520214, 520225, 520310, 520321, 520332, 520343, 520354, 520365, 520376, 520380, 520391, 520402, 520413, 520424, 520435, 520446, 520450, 520461, 520472, 520483, 520494, 520505, 520516, 520520, 520531, 520542, 520553, 520564, 520575, 520586, 520590, 520601, 520612, 520623, 520634, 520645, 520656, 520660, 520671, 520682, 520693, 520704, 520715, 520726, 520730, 520741, 520752, 520763, 520774, 520785, 520796, 520800, 520811, 520822, 520833, 520844, 520855, 520866, 520870, 520881, 520892, 520903, 520914, 520925, 520936, 520940, 520951, 520962, 520973, 520984, 520995, 521006, 521010, 521021, 521032, 521043, 521054, 521065, 521076, 521080, 521091, 521102, 521113, 521124, 521135, 521146, 521150, 521161, 521172, 521183, 521194, 521205, 521216, 521220, 521231, 521242, 521253, 521264, 521275, 521286, 521290, 521301, 521312, 521323, 521334, 521345, 521356, 521360, 521371, 521382, 521393, 521404, 521415, 521426, 521430, 521441, 521452, 521463, 521474, 521485, 521496, 521500, 521511, 521522, 521533, 521544, 521555, 521566, 521570, 521581, 521592, 521603, 521614, 521625, 521636, 521640, 521651, 521662, 521673, 521684, 521695, 521706, 521710, 521721, 521732, 521743, 521754, 521765, 521776, 521780, 521791, 521802, 521813, 521824, 521835, 521846, 521850, 521861, 521872, 521883, 521894, 521905, 521916, 521920, 521931, 521942, 521953, 521964, 521975, 521986, 521990, 522001, 522012, 522023, 522034, 522045, 522056, 522060, 522071, 522082, 522093, 522104, 522115, 522126, 522130, 522141, 522152, 522163, 522174, 522185, 522196, 522200, 522211, 522222, 522233, 522244, 522255, 522266, 522270, 522281, 522292, 522303, 522314, 522325, 522336, 522340, 522351, 522362, 522373, 522384, 522395, 522406, 522410, 522421, 522432, 522443, 522454, 522465, 522476, 522480, 522535, 522550, 522572, 522583, 522594, 522605, 522616, 522620, 522631, 522642, 522653, 522664, 522675, 522686, 522734, 522745, 522756, 522760, 522771, 522782, 522793, 522804, 522815, 522826, 522830, 522841, 522852, 522863, 522874, 522885, 522896, 522900, 522911, 522922, 522933, 522944, 522955, 522966, 522970, 522981, 523014, 523025, 523036, 523040, 523051, 523062, 523073, 523084, 523095, 523106, 523110, 523121, 523132, 523143, 523154, 523165, 523176, 523180, 523191, 523202, 523213, 523224, 523235, 523246, 523250, 523261, 523272, 523283, 523294, 523305, 523316, 523320, 523331, 523342, 523353, 523364, 523375, 523386, 523390, 523401, 523412, 523423, 523434, 523445, 523456, 523460, 523471, 523482, 523493, 523504, 523515, 523526, 523530, 523541, 523552, 523563, 523574, 523585, 523596, 523600, 523611, 523622, 523633, 523644, 523655, 523666, 523670, 523681, 523692, 523703, 523714, 523725, 523736, 523740, 523751, 523762, 523773, 523784, 523795, 523806, 523810, 523821, 523832, 523843, 523854, 523865, 523876, 523880, 523891, 523902, 523913, 523924, 523935, 523946, 523950, 523961, 523972, 523983, 523994, 524005, 524016, 524020, 524031, 524042, 524053, 524064, 524075, 524086, 524090, 524101, 643451, 643462, 653656, 653660, 770394, 770405, 770416, 770420 |
| Rheumatoid arthritis | | **ICD-10:** M05, M06,  **ATC:** L04AA13, L04AA24  **Medical procedure code:** 478030, 478041 |
| Connective tissue disease | | **ICD-10:** M05.0, M05.1, M05.2**,** M05.3, M05.8, M05.9, M06.0, M06.3, M06.9, M30, M31, M32, M33, M34, M35, M36 |
| Arthritis (any type) | | **ICD-10:** L40.5, M02.3, M05, M06, M08, M13.0, M13.1, M15-M19, M45, M46.1, M46.8, M46.9  **ATC**: L04AA13, L04AA24  **Medical procedure code:** 478030, 478041 |
| Gout or other crystal-induced arthropathy | | **ICD-10:** M10, M11, M1A  **ATC:** M04A |
| Musculoskeletal problems | | **ICD-10:** M02, M07, M12.0, M12.1, M12.2, M12.3, M12.4, M12.8, M12.9, M13, M14, M24.0, M24.3, M24.6, M24.7, M24.8, M24.9, M25, M36.1, M36.2, M36.3, M36.4, M45, M46.0, M46.1, M46.4, M46.8, M46.9, M47, M48, M49, M50, M51, M53, M54, M80, M81, M84.3, M84.4, M84.5, M84.6, Z87.31, Z87.39 |
| Dementia | | **ICD-10:** A81.0, F01, F02, F03, F05, G30, G31.0, G31.83, G31.85  **ATC**: N06D |
| Cognitive deterioration (without dementia) | | **ICD-10:** G31.1, G31.84, G31.89, G31.9, R41.81 |
| Parkinson’s disease | | **ICD-10:** G20, G21, G23.1, G31.83, G31.85, G90.3  **ATC:** N04AB, N04AC, N04B |
| Depression | | **ICD-10:** F06.31, F06.32, F30, F31, F32, F33, F34.1, F43.21, F43.23, F53.0  **ATC**: N06A |
| Schizophrenia & paranoia | | **ICD-10:** F06.0, F06.2, F20, F22, F23, F24, F28, F29 |
| Chronic skin ulcer | | **ICD-10:** E08.621, E08.622, E09.621, E09.622, E10.621, E10.622, E11.621, E11.622, E13.621, E13.622, L89, L97, L98.4  **Medical procedure code:** 114074, 114085 |
| HIV/AIDS | | **ICD-10:** B20, B97.35, Z21  **ATC:** J05AE01 , J05AE03, J05AE04, J05AE05, J05AE07, J05AE08, J05AE09, J05AE10, J05AF01, J05AF02, J05AF03, J05AF04, J05AF06, J05AF09, J05AF11, J05AF12, J05AG, J05AJ, J05AR, J05AX07, J05AX09, J05AX29 |
| Skin and soft tissue infections | | **ICD-10:** A06.7, A28.1, A31.1, A43.1, A46, A50.06, A51.3, A60.1, A63.0, A66.2, L00, L01, L02, L03, L04, L05, L08, L88  **Medical procedure code:** 145552, 145563, 145574, 145585, 220253, 220264, 244650, 244661 |
| Mycosis(10) | | **ICD-10:** B35-B49  **ATC:** D01A, D01BA02 |
| Systemic lupus erythematosus | | **ICD-10:** M32 |
| Vasculitis | | **ICD-10:** M05.2, I77.6  **Medical procedure code:** 552532, 552543 |
| Sarcoidosis | | **ICD-10:** D86 |
| Inflammatory bowel disease | | **ICD-10:** K50, K51, K52  **ATC:** A07EA, A07EC02, A07EC03, A07EC04 |
| Chronic rejection after solid-organ transplantation | | **ICD-10:** T86 |
| Malnutrition | | **ICD-10:** E40-E46 |
| **DISEASE SEVERITY-RELATED VARIABLES** | | |
| Antibiotics | | **ATC:** J01 |
| Respiratory failure | | **ICD-10:** J96 |
| Oxygen therapy | | **ATC:** V03AN01  **Medical procedure code:** 355095, 355106, 355110, 355121, 523795, 523806, 751030, 751052, 755952, 757455, 757470, 757831, 760642, 797355 |
| Non-invasive ventilation | | **ICD-10-PCS:** 5A09357, 5A09358, 5A09457, 5A09458, 5A09557, 5A09558 |
| Invasive ventilation | | **ICD-10-PCS:** 5A0935Z, 5A0945Z, 5A0955Z, 0BH17EZ, 0BH18EZ, 0BH13EZ |
| Admission to intensive care unit | | **Medical procedure code:** 211223, 211245, 211945, 518081, 518103, 557900, 557944, 557981 |
| **ANEMIA TREATMENT (≤1 year before index date)(4)** | | |
| Iron | | **ATC:** B03A |
| Vitamin B12 | | **ATC:** B03AE01, B03BA |
| Folic acid | | **ATC:** B03BB, B03AE01, B03AE02, B03AD |
| Erythroid stimulating agents | | **ATC:** B03XA01, B03XA02, B03XA03 |
| **CLINICAL RISK SCORES(4,5)** | | |
| John Hopkins Claims-based Frailty Indicator(11-15) | | - **Impaired mobility**: beta coefficient 1.24 (definition mentioned above: ‘Impaired mobility’)  - **Depression**: beta coefficient 0.54 (definition mentioned above: ‘Depression’)  - **Heart failure**: beta coefficient 0.50 (definition mentioned above: ‘Heart failure’)  - **Parkinson’s disease**: beta coefficient 0.50 (definition mentioned above: ‘Parkinson’s disease’)  - **White race**: beta coefficient -0.49: not available  - **Arthritis (any type):** beta coefficient 0.43 (definition mentioned above: ‘Arthritis’)  - **Cognitive impairment**: beta coefficient 0.33 (combination of definitions mentioned above: ‘Dementia’ and ‘Cognitive deterioration’)   - **ICD-10:** A81.0, F01, F02, F03, F05, G30, G31.0, G31.1, G31.83, G31.84, G31.85, G31.89, G31.9, R41.81 - **ATC:** N06D   - **Charlson comorbidity index** **(> 0)**: beta coefficient 0.31  - **Stroke**: beta coefficient 0.28 (definition mentioned above: ‘Stroke’)  - **Paranoia**: beta coefficient 0.24 (definition mentioned above: ‘Schizophrenia & paranoia’)  - **Chronic skin ulcer**: beta coefficient 0.23 (definition mentioned above: ‘Chronic skin ulcer’)  - **Pneumonia**: beta coefficient 0.21   - **ICD-10:** A01.03, A02.22, A37.01, A37.11, A37.81, A37.91, A50.04, A54.84, B01.2, B05.2, B06.81, B77.81, J09.X1, J09.X2, J09.X3, J10.0, J11.0, J12, J13, J14, J15, J16, J17, J18, J84.11, J84.2, J85.1, J95.851, Z87.01   - **Male sex**: beta coefficient -0.19  - **Skin and soft tissue infection**: beta coefficient 0.18 (definition mentioned above: ‘Skin and soft tissue infection’)  - **Mycoses**: beta coefficient 0.14 (definition mentioned above: ‘Mycosis’)  - **Age (for every 1 year increase)**: beta coefficient 0.09  - **Admission in past 6 months**: beta coefficient 0.09  - **Gout or other crystal-induced arthropathy**: beta coefficient 0.08 (definition mentioned above: ‘Gout or other crystal-induced arthropathy’)  - **Falls**: beta coefficient 0.08 (definition mentioned above: ‘History of falling’)  - **Musculoskeletal problems**: beta coefficient 0.05 (definition mentioned above: ‘Musculoskeletal problems’)  - **Urinary tract infection**: beta coefficient 0.05 (definition mentioned above: ‘Urinary tract infection) |
| Charlson Comorbidity Index(16-20) | | - **Myocardial infarction:** 1 point (definition mentioned above: ‘Myocardial infarction’)  - H**eart failure**: 1 point (definition mentioned above: ‘Heart failure’)  - **Peripheral vascular disease**: 1 point (definition mentioned above: ‘Peripheral vascular disease)  - **Cerebrovascular disease:** 1 point (definition mentioned above: ‘Cerebrovascular disease’)  - **Dementia**: 1 point (definition mentioned above: ‘Dementia’)  - **Connective tissue disease**: 1 point (definition mentioned above: ‘Connective tissue disease’)  - **Peptic ulcer disease**: 1 point (definition mentioned above: ‘Peptic ulcer disease’)  - **Mild liver disease**: 1 point (definition mentioned above: ‘Mild liver disease’)  - **Diabetes without chronic complications**: 1 point (definition mentioned above: ‘Diabetes’ and/or ‘Diabetes without vascular complications’)  - **Diabetes with chronic complications**: 2 point (definition mentioned above: ‘Diabetes with vascular complications)  - **Hemiplegia or paraplegia**: 2 points (definition mentioned above: ‘Hemiplegia/paraplegia)  - **Renal disease:** 2 points (definition mentioned above: ‘Chronic kidney disease’)  - **Any malignancy, including leukemia and lymphoma:** 2 points (definition mentioned above: ‘Any malignancy, including leukemia and lymphoma’)  - **Moderate or severe liver disease:** 3 points (definition mentioned above: ‘Moderate or severe liver disease’)  - **Metastatic solid tumor:** 6 points (definition mentioned above: ‘Metastatic cancer’)  - **AIDS/HIV:** 6 points (definition mentioned above: ‘HIV’)  - **Age:**   - <50 years: 0 points - 50-59 years: 1 point - 60-69 years: 2 points - 70-79 years: 3 points - ≥80 years: 4 points   *The following comorbid conditions were mutually exclusive: diabetes with chronic complications and diabetes without chronic complications; mild liver disease and moderate or severe liver disease; and any malignancy and metastatic solid tumor. Patients with diabetes without specific ICD-codes linked to diabetes with or without chronic complications were considered as patients with diabetes without chronic complications. As it is suspected that all patients included in our study cohort have chronic pulmonary disease, this factor was not taken into account to calculate the Charlson Comorbidity Index.* |
| **OUTCOMES** | | |
| Mortality | | All-cause mortality |
| Hospitalized COPD exacerbation | | Hospital admissions or emergency department visits with a primary diagnosis code of (acute) COPD exacerbation, COPD with acute lower respiratory infection (**ICD-10:** J44.0 or J44.1)  OR  with a primary diagnosis of chronic lower respiratory disease (**ICD-10:** J40-J47) or respiratory failure (**ICD-10:** J96), combined with a secondary diagnosis code of (acute) COPD exacerbation, COPD with acute lower respiratory infection (**ICD-10:** J44.0 or J44.1).  Hospitalizations with a concomitant pneumonia diagnosis were excluded (**ICD-10:** A01.03, A02.22, A37.01, A37.11, A37.81, A37.91, A50.04, A54.84, B01.2, B05.2, B06.81, B77.81, J09.X1, J09.X2, J09.X3, J10.0, J11.0, J12, J13, J14, J15, J16, J17, J18, J84.11, J84.2, J85.1, J95.851). |

## eTable 3: Cox proportional hazards models of post-discharge mortality using inverse probability of treatment weighting for iron therapy, including interaction terms with anemia subtypes

|  | | aHR (95% CI) | P-value |
| --- | --- | --- | --- |
| Anemia subtype (ref: no anemia) | |  |  |
|  | Hematologic anemia | 2.73 (2.26, 3.30) | **<0.001** |
|  | Anemia of chronic disease | 1.98 (1.77, 2.21) | **<0.001** |
|  | Nutritional anemia | 1.70 (1.52, 1.90) | **<0.001** |
|  | Other types of anemia | 0.94 (0.77, 1.15) | 0.537 |
| Iron | | 1.38 (1.24, 1.53) | **<0.001** |
| **Hematologic anemia x iron** | | **0.68 (0.49, 0.95)** | **0.025** |
| **Anemia of chronic disease x iron** | | **0.81 (0.67, 0.98)** | **0.034** |
| **Nutritional anemia x iron** | | **0.65 (0.55, 0.78)** | **<0.001** |
| Other type of anemia x iron | | 0.88 (0.57, 1.38) | 0.586 |

Propensity scores were estimated using logistic regression separately for each anemia treatment. Covariates included demographics (age, sex, socio-economic status, (ever-)smoking, and weight), comorbidities (overall anemia diagnosis, frailty, cancer, malnutrition, chronic kidney disease, heart failure, diabetes mellitus, arrythmia and hypertension), indicators of disease severity (oxygen therapy, non-invasive and invasive ventilation, respiratory failure, antibiotic therapy, intensive care unit admission, and length of hospital stay), as well as other anemia-related treatments.

*aHR = adjusted hazard ratio; CI = Confidence interval*

## eTable 4: Cox proportional hazards models of post-discharge mortality using inverse probability of treatment weighting for vitamin B12 therapy, including interaction terms with anemia subtypes

|  | | aHR (95% CI) | P-value |
| --- | --- | --- | --- |
| Anemia subtype (ref: no anemia) | |  |  |
|  | Hematologic anemia | 2.41 (2.00, 2.91) | **<0.001** |
|  | Anemia of chronic disease | 2.29 (2.06, 2.54) | **<0.001** |
|  | Nutritional anemia | 1.71 (1.56, 1.89) | **<0.001** |
|  | Other types of anemia | 1.01 (0.81, 1.25) | 0.934 |
| Vitamin B12 | | 1.50 (1.19, 1.88) | **<0.001** |
| Hematologic anemia x vitamin B12 | | 0.80 (0.44, 1.46) | 0.474 |
| Anemia of chronic disease x vitamin B12 | | 0.70 (0.44, 1.12) | 0.136 |
| **Nutritional anemia x vitamin B12** | | **0.65 (0.45, 0.95)** | **0.028** |
| Other type of anemia x vitamin B12 | | 0.52 (0.06, 4.46) | 0.549 |

Propensity scores were estimated using logistic regression separately for each anemia treatment. Covariates included demographics (age, sex, socio-economic status, (ever-)smoking, and weight), comorbidities (overall anemia diagnosis, frailty, cancer, malnutrition, chronic kidney disease, heart failure, diabetes mellitus, arrythmia and hypertension), indicators of disease severity (oxygen therapy, non-invasive and invasive ventilation, respiratory failure, antibiotic therapy, intensive care unit admission, and length of hospital stay), as well as other anemia-related treatments.

*aHR = adjusted hazard ratio; CI = Confidence interval*

## eTable 5: Cox proportional hazards models of post-discharge mortality using inverse probability of treatment weighting for folic acid therapy, including interaction terms with anemia subtypes

|  | | aHR (95% CI) | P-value |
| --- | --- | --- | --- |
| Anemia subtype (ref: no anemia) | |  |  |
|  | Hematologic anemia | 3.34 (2.80, 3.98) | **<0.001** |
|  | Anemia of chronic disease | 2.29 (2.07, 2.52) | **<0.001** |
|  | Nutritional anemia | 1.81 (1.65, 1.98) | **<0.001** |
|  | Other types of anemia | 1.07 (0.86, 1.34) | 0.515 |
| Folic acid | | 1.10 (1.01, 1.20) | **0.024** |
| **Hematologic anemia x folic acid** | | **0.62 (0.45, 0.86)** | **0.004** |
| Anemia of chronic disease x folic acid | | 0.97 (0.80, 1.17) | 0.716 |
| Nutritional anemia x folic acid | | 0.87 (0.74, 1.03) | 0.105 |
| Other type of anemia x folic acid | | 0.95 (0.63, 1.45) | 0.816 |

Propensity scores were estimated using logistic regression separately for each anemia treatment. Covariates included demographics (age, sex, socio-economic status, (ever-)smoking, and weight), comorbidities (overall anemia diagnosis, frailty, cancer, malnutrition, chronic kidney disease, heart failure, diabetes mellitus, arrythmia and hypertension), indicators of disease severity (oxygen therapy, non-invasive and invasive ventilation, respiratory failure, antibiotic therapy, intensive care unit admission, and length of hospital stay), as well as other anemia-related treatments.

*aHR = adjusted hazard ratio; CI = Confidence interval*

## eTable 6: Cox proportional hazards models of post-discharge mortality using inverse probability of treatment weighting for ESA therapy, including interaction terms with anemia subtypes

|  | | aHR (95% CI) | P-value |
| --- | --- | --- | --- |
| Anemia subtype (ref: no anemia) | |  |  |
|  | Hematologic anemia | 2.43 (1.81, 3.26) | **<0.001** |
|  | Anemia of chronic disease | 1.77 (1.51, 2.06) | **<0.001** |
|  | Nutritional anemia | 1.40 (1.19, 1.64) | **<0.001** |
|  | Other types of anemia | 0.73 (0.55, 0.97) | **0.031** |
| ESA | | 1.90 (1.56, 2.33) | **<0.001** |
| Hematologic anemia x ESA | | 0.64 (0.41, 1.02) | 0.062 |
| **Anemia of chronic disease x ESA** | | **0.63 (0.46, 0.85)** | **0.003** |
| **Nutritional anemia x ESA** | | **0.56 (0.32, 0.97)** | **0.038** |
| Other type of anemia x ESA | | - | - |

Propensity scores were estimated using logistic regression separately for each anemia treatment. Covariates included demographics (age, sex, socio-economic status, (ever-)smoking, and weight), comorbidities (overall anemia diagnosis, frailty, cancer, malnutrition, chronic kidney disease, heart failure, diabetes mellitus, arrythmia and hypertension), indicators of disease severity (oxygen therapy, non-invasive and invasive ventilation, respiratory failure, antibiotic therapy, intensive care unit admission, and length of hospital stay), as well as other anemia-related treatments.

*ESA = erythroid stimulating agent*

*aHR = adjusted hazard ratio; CI = Confidence interval*

# Supplemental Figures

## eFigure 1: Overview of the study design

eFigure 1: Study design of the longitudinal cohort study

## eFigure 2: Flowchart of study population.

eFigure 2: Flowchart of inclusion of study population

*ATC: Anatomical Therapeutic Chemical; IMA: InterMutualistic Agency*

## eFigure 3: Love plots

A) Love plot showing covariate balance before and after inverse probability of treatment weighting for iron therapy

B) Love plot showing covariate balance before and after inverse probability of treatment weighting for vitamin B12 therapy

C) Love plot showing covariate balance before and after inverse probability of treatment weighting for folic acid therapy

D) Love plot showing covariate balance before and after inverse probability of treatment weighting for erythroid stimulating agent therapy

## eFigure 4: Kaplan-Meier curve comparing patients with and without anemia

eFigure 4: Kaplan-Meier curve comparing patients with and without anemia, with number at risk specified

## eFigure 5: Logistic regression analyses for the association between anemia and in-hospital mortality in COPD patients hospitalized for an exacerbation

eFigure 5: Logistic regression analyses for the association between anemia and in-hospital mortality in COPD patients hospitalized for an exacerbation

Panel A. Overall anemia. Panel B. Anemia classified into subtypes.
Model 1: adjusted for demographics (age, sex, SES, (ever-)smoking and weight).
Model 2: model 1 + adjustment for CCI.
*OR = odds ratio*

Among anemic patients (6,706 (19.2%) of 34,864 hospitalized for AECOPD, including patients who died during hospitalization), 3,279 (48.9%) had nutritional anemia, 2,148 (32.0%) ACD, 632 (9.4%) hematologic anemia, and 647 (9.6%) other types.

## eFigure 6 sensitivity analyses: Cox analyses for the association between anemia and post-discharge mortality adjusted for comorbidities

eFigure 6. Sensitivity analyses: Cox analyses for the association between anemia and post-discharge mortality in COPD patients hospitalized for COPD exacerbation.

Panel A. Anemia of any type. Panel B. Anemia classified into subtypes.
Sensitivity analysis: adjusted for comorbidities (frailty, cancer, malnutrition, chronic kidney disease, heart failure, diabetes mellitus, arrythmia, and hypertension)
*CI = Confidence interval; HR = Hazard ratio*

## eFigure 7 sensitivity analyses: Cox analyses for the association between anemia subtypes and post-discharge mortality in COPD patients hospitalized for an exacerbation (without reclassification of other/unspecified anemia)

eFigure 7: Cox analyses for the association between anemia subtypes and post-discharge mortality in COPD patients hospitalized for an exacerbation (without reclassification of other/unspecified anemia).
Model 1: adjusted for demographics (age, sex, SES, (ever-)smoking and weight).
Model 2: Model 1 + adjustment for age-adjusted Charlson Comorbidity Index.
*CI = Confidence interval; HR = Hazard ratio*

Without reclassification, among anemic patients, 2,640 (44.3%) had nutritional anemia, 719 (12.1%) ACD, 486 (8.2%) hematologic anemia, and 2,111 (35.4%) other/unspecified anemia.

## eFigure 8 sensitivity analyses: Cox analyses for the association between anemia subtypes and readmission for severe exacerbation in COPD patients hospitalized for an exacerbation (without reclassification of other/unspecified anemia)

eFigure 8: Cox analyses for the association between anemia subtypes and readmission for severe exacerbation in COPD patients hospitalized for an exacerbation (without reclassification of other/unspecified anemia).
Model 1: adjusted for demographics (age, sex, SES, (ever-)smoking and weight).
Model 2: Model 1 + adjustment for age-adjusted Charlson Comorbidity Index.
*CI = Confidence interval; HR = Hazard ratio*

Without reclassification, among anemic patients, 2,640 (44.3%) had nutritional anemia, 719 (12.1%) ACD, 486 (8.2%) hematologic anemia, and 2,111 (35.4%) other/unspecified anemia.

## eFigure 9 sensitivity analyses: Logistic regression analyses for the association between anemia and in-hospital mortality in COPD patients hospitalized for an exacerbation (without reclassification of other/unspecified anemia)

eFigure 9: Logistic regression analyses for the association between anemia and in-hospital mortality in COPD patients hospitalized for an exacerbation (without reclassification of other/unspecified anemia)
Model 1: adjusted for demographics (age, sex, SES, (ever-)smoking and weight).
Model 2: Model 1 + adjustment for age-adjusted Charlson Comorbidity Index.
*CI = Confidence interval; HR = Hazard ratio*

Without reclassification, among anemic patients, 2,935 (43.8%) had nutritional anemia, 827 (12.3%) ACD, 550 (8.2%) hematologic anemia, and 2,394 (35.7%) other/unspecified anemia.

## eFigure 10 sensitivity analyses: Cox analyses for the association between anemia subtypes and post-discharge mortality in COPD patients hospitalized for an exacerbation, without reclassification and excluding other/unspecified anemia

eFigure 10: Cox analyses for the association between anemia subtypes and post-discharge mortality in COPD patients hospitalized for an exacerbation, without reclassification and excluding other/unspecified anemia.
Model 1: adjusted for demographics (age, sex, SES, (ever-)smoking and weight).
Model 2: Model 1 + adjustment for age-adjusted Charlson Comorbidity Index.
*CI = Confidence interval; HR = Hazard ratio*

In this analysis, 2,111 patients with other/unspecified anemia were excluded. Without reclassification, among 3,845 clearly defined anemic patients, 2,640 (68.7%) had nutritional anemia, 719 (18.7%) ACD and 486 (12.6%) hematologic anemia.

## eFigure 11 sensitivity analyses: Cox analyses for the association between anemia subtypes and readmission for severe exacerbation in COPD patients hospitalized for an exacerbation, without reclassification and excluding other/unspecified anemia

eFigure 11: Cox analyses for the association between anemia subtypes and readmission for severe exacerbation in COPD patients hospitalized for an exacerbation, without reclassification and excluding other/unspecified anemia.
Model 1: adjusted for demographics (age, sex, SES, (ever-)smoking and weight).
Model 2: Model 1 + adjustment for age-adjusted Charlson Comorbidity Index.
*CI = Confidence interval; HR = Hazard ratio*

In this analysis, 2,111 patients with other/unspecified anemia were excluded. Without reclassification, among 3,845 clearly defined anemic patients, 2,640 (68.7%) had nutritional anemia, 719 (18.7%) ACD and 486 (12.6%) hematologic anemia.

## eFigure 12 sensitivity analyses: Logistic regression analyses for the association between anemia and in-hospital mortality in COPD patients hospitalized for an exacerbation, without reclassification and excluding other/unspecified anemia

eFigure 12: Logistic regression analyses for the association between anemia and in-hospital mortality in COPD patients hospitalized for an exacerbation, without reclassification and excluding other/unspecified anemia.
Model 1: adjusted for demographics (age, sex, SES, (ever-)smoking and weight).
Model 2: Model 1 + adjustment for age-adjusted Charlson Comorbidity Index.
*CI = Confidence interval; HR = Hazard ratio*

In this analysis, 2,394 patients with other/unspecified anemia were excluded. Without reclassification, among 4,312 clearly defined anemic patients, 2,935 (68.1%) had nutritional anemia, 827 (19.2%) ACD, and 550 (12.8%) hematologic anemia.

## eFigure 13 sensitivity analyses: Cox analyses for the association between anemia subtypes and readmission for severe exacerbation in COPD patients hospitalized for an exacerbation using Fine-Gray subdistribution hazard model (Model 2)

eFigure 13: Cox analyses for the association between anemia subtypes and readmission for severe exacerbation in COPD patients hospitalized for an exacerbation using Fine-Gray subdistribution hazard model (Model 2)

Panel A. Anemia of any type. Panel B. Anemia classified into subtypes.
Adjusted for demographics (age, sex, SES, (ever-)smoking and weight) and for age-adjusted Charlson Comorbidity Index.
*CI = Confidence interval; SHR = subdistribution hazard ratio*

# References

1.	Maetens A, De Schreye R, Faes K, Houttekier D, Deliens L, Gielen B, et al. Using linked administrative and disease-specific databases to study end-of-life care on a population level. BMC Palliat Care. 2016;15(1):86.

2.	IMA/AIM. InterMutualistic Agency (IMA/AIM) 2024 [Available from: <https://ima-aim.be/>.

3.	Belgian Ministry of Health (FOD Volksgezondheid). The minimal hospital dataset 2024 [Available from: <https://www.health.belgium.be/nl/gezondheid/organisatie-van-de-gezondheidszorg/ziekenhuizen/registratiesystemen/mzg>.

4.	WHO Collaborating Centre for Drug Statistics Methodology. Available from: <https://www.whocc.no/>. Accessed 25 November 2021.

5.	The International Classification of Diseases (ICD), Clinical Modification. Available from: <https://www.cdc.gov/nchs/icd/index.htm>. Accessed 25 November 2021.

6.	RIZIV/INAMI (Rijksinstituut voor ziekte- en invaliditeitsverzekering/Institut national d'assurance maladie-invalidité) medical procedure codes for claims of ambulatory and hospital care. Available from: <https://www.riziv.fgov.be/nl/nomenclatuur/Paginas/default.aspx> (in Dutch/French). Accessed 25 November 2021.

7.	Lorenzoni G, Baldi I, Soattin M, Gregori D, Buja A. A Systematic Review of Case-Identification Algorithms Based on Italian Healthcare Administrative Databases for Three Relevant Diseases of the Cardiovascular System: Hypertension, Heart Failure, and Congenital Heart Diseases. Epidemiol Prev. 2019;43(4 Suppl 2):51-61.

8.	Chakhtoura M, Haber R, Ghezzawi M, Rhayem C, Tcheroyan R, Mantzoros CS. Pharmacotherapy of obesity: an update on the available medications and drugs under investigation. EClinicalMedicine. 2023;58:101882.

9.	Kranz J, Schmidt S, Lebert C, Schneidewind L, Mandraka F, Kunze M, et al. The 2017 Update of the German Clinical Guideline on Epidemiology, Diagnostics, Therapy, Prevention, and Management of Uncomplicated Urinary Tract Infections in Adult Patients. Part II: Therapy and Prevention. Urol Int. 2018;100(3):271-8.

10.	Thompson GR, 3rd, Le T, Chindamporn A, Kauffman CA, Alastruey-Izquierdo A, Ampel NM, et al. Global guideline for the diagnosis and management of the endemic mycoses: an initiative of the European Confederation of Medical Mycology in cooperation with the International Society for Human and Animal Mycology. Lancet Infect Dis. 2021;21(12):e364-e74.

11.	Kundi H, Coskun N, Yesiltepe M. Association of entirely claims-based frailty indices with long-term outcomes in patients with acute myocardial infarction, heart failure, or pneumonia: a nationwide cohort study in Turkey. The Lancet Regional Health -Europe. 2021;10:100183.

12.	Segal JB, Chang HY, Du Y, Walston JD, Carlson MC, Varadhan R. Development of a Claims-based Frailty Indicator Anchored to a Well-established Frailty Phenotype. Med Care. 2017;55(7):716-22.

13.	Segal JB, Huang J, Roth DL, Varadhan R. External validation of the claims-based frailty index in the national health and aging trends study cohort. Am J Epidemiol. 2017;186(6):745-7.

14.	Grymonprez M, Petrovic M, De Backer TL, Steurbaut S, Lahousse L. Impact of frailty on the effectiveness and safety of non-vitamin K antagonist oral anticoagulants (NOACs) in patients with atrial fibrillation: a nationwide cohort study. Eur Heart J Qual Care Clin Outcomes. 2024;10(1):55-65.

15.	Le Pogam M-A, Seematter-Bagnoud L, Niemi T, Assouline D, Gross N, Trächsel B, et al. Development and validation of a knowledge-based score to predict Fried's frailty phenotype across multiple settings using one-year hospital discharge data: The electronic frailty score. eClinicalMedicine. 2022;44:101260.

16.	Quan H, Sundararajan V, Halfon P, Fong A, Burnand B, Luthi JC, et al. Coding algorithms for defining comorbidities in ICD-9-CM and ICD-10 administrative data. Med Care. 2005;43(11):1130-9.

17.	Sundararajan V, Henderson T, Perry C, Muggivan A, Quan H, Ghali WA. New ICD-10 version of the Charlson comorbidity index predicted in-hospital mortality. J Clin Epidemiol. 2004;57(12):1288-94.

18.	Charlson M, Szatrowski TP, Peterson J, Gold J. Validation of a combined comorbidity index. J Clin Epidemiol. 1994;47(11):1245-51.

19.	Grymonprez M, De Backer TL, Bertels X, Steurbaut S, Lahousse L. Long-term comparative effectiveness and safety of dabigatran, rivaroxaban, apixaban and edoxaban in patients with atrial fibrillation: A nationwide cohort study. Front Pharmacol. 2023;14:1125576.

20.	Yang CC, Fong Y, Lin LC, Que J, Ting WC, Chang CL, et al. The age-adjusted Charlson comorbidity index is a better predictor of survival in operated lung cancer patients than the Charlson and Elixhauser comorbidity indices. Eur J Cardiothorac Surg. 2018;53(1):235-40.
